# Economic implications of novel regimens for tuberculosis treatment in three high-burden countries: a modelling analysis

**DOI:** 10.1016/S2214-109X(24)00088-3

**Published:** 2024-05-16

**Authors:** Theresa S Ryckman, Samuel G Schumacher, Christian Lienhardt, Sedona Sweeney, David W Dowdy, Fuad Mirzayev, Emily A Kendall

**Affiliations:** a Division of Infectious Diseases, Department of Medicine, Johns Hopkins University School of Medicine, Baltimore, MD, USA; b Global TB Programme, WHO, Geneva, Switzerland; c Institut de Recherche pour le Développement, Université de Montpellier, Montpellier, France; d Department of Global Health and Development, London School of Hygiene & Tropical Medicine, London, UK; e Department of Epidemiology, Johns Hopkins Bloomberg School of Public Health, Baltimore, MD, USA

## Abstract

**Background:**

With numerous trials investigating novel drug combinations to treat tuberculosis, we aimed to evaluate the extent to which future improvements in tuberculosis treatment regimens could offset potential increases in drug costs.

**Methods:**

In this modelling analysis, we used an ingredients-based approach to estimate prices at which novel regimens for rifampin-susceptible and rifampin-resistant tuberculosis treatment would be cost-neutral or cost-effective compared with standards of care in India, the Philippines, and South Africa. We modelled regimens meeting targets set in the WHO's 2023 Target Regimen Profiles (TRPs). Our decision-analytical model tracked cohorts of adults initiating rifampin-susceptible or rifampin-resistant tuberculosis treatment, simulating their health outcomes and costs accumulated during and following treatment under standard-of-care and novel regimen scenarios. Price thresholds included short-term cost-neutrality (considering only savings accrued during treatment), medium-term cost-neutrality (additionally considering savings from averted retreatments and secondary cases), and cost-effectiveness (incorporating willingness-to-pay for improved health outcomes).

**Findings:**

Total medium-term costs per person treated using standard-of-care regimens were estimated at US$450 (95% uncertainty interval 310–630) in India, $560 (350–860) in the Philippines, and $730 (530–1090) in South Africa for rifampin-susceptible tuberculosis (current drug costs $46) and $2100 (1590–2810) in India, $2610 (2090–3280) in the Philippines, and $3790 (3090–4630) in South Africa for rifampin-resistant tuberculosis (current drug costs $432). A rifampin-susceptible tuberculosis regimen meeting the optimal targets defined in the TRPs could be cost-neutral in the short term at drug costs of $140 (90–210) per full course in India, $230 (130–380) in the Philippines, and $280 (180–460) in South Africa. For rifampin-resistant tuberculosis, short-term cost-neutral thresholds were higher with $930 (720–1230) in India, $1180 (980–1430) in the Philippines, and $1480 (1230–1780) in South Africa. Medium-term cost-neutral prices were approximately $50–100 higher than short-term cost-neutral prices for rifampin-susceptible tuberculosis and $250–550 higher for rifampin-resistant tuberculosis. Health system cost-neutral prices that excluded patient-borne costs were 45–70% lower (rifampin-susceptible regimens) and 15–50% lower (rifampin-resistant regimens) than the cost-neutral prices that included patient costs. Cost-effective prices were substantially higher. Shorter duration was the most important driver of medium-term savings with novel regimens, followed by ease of adherence.

**Interpretation:**

Improved tuberculosis regimens, particularly shorter regimens or those that facilitate better adherence, could reduce overall costs, potentially offsetting higher prices.

**Funding:**

WHO.

## Introduction

Tuberculosis caused an estimated 10·6 million illnesses and 1·6 million deaths in 2021.^1^ Both the WHO End tuberculosis Strategy and the Stop Tuberculosis Partnership Global Plan to End Tuberculosis name new treatment regimens for rifampin-susceptible and rifampin-resistant tuberculosis as crucial components to reaching global targets, such as drastically reducing tuberculosis deaths.^2^, ^3^

Over the past decade, several important advances in tuberculosis therapeutics have been made, prompting changes to global policy recommendations.^4^, ^5^ For rifampin-resistant-tuberculosis, a regimen of bedaquiline, pretomanid, linezolid, and moxifloxacin (BPaLM), represents a substantial improvement over the previous standards of care in terms of duration, efficacy, safety profile, and costs.^6^, ^7^, ^8^, ^9^ Future rifampin-resistant tuberculosis regimens therefore need to be evaluated against this new benchmark. For rifampin-susceptible tuberculosis, efforts to shorten and otherwise improve upon the 6-month standards of care are starting to come to fruition, as demonstrated by recent clinical trial successes of 4-month regimens^10^, ^11^ and increasing evidence that many patients can be cured with shorter treatment courses.^12^, ^13^ Several new drugs and novel drug combinations in the developmental pipeline show promise for further improvements to treatment outcomes.^14^, ^15^

**Research in context**

**Evidence before this study**

Previous modelling analysis projected the health effects of novel tuberculosis treatment regimens meeting target profiles but did not incorporate costs. Studies have also evaluated the cost-effectiveness of specific regimens that are now in widespread use. To identify relevant cost-related studies of future novel regimens, we searched PubMed using the following search terms for the titles and abstracts: (tuberculosis OR tuberculosis) AND (cost OR economic) AND (novel OR improved) AND (model OR analysis OR estimate) AND (treatment OR therapeutic OR regimen). We did the search with no language restrictions on Jan 3, 2024 and restricted our search to human studies published between Jan 1, 2010 and Dec 31, 2023. This search yielded 156 published studies. Several studies analysed the cost or cost-effectiveness of existing regimens and programmes to treat tuberculosis disease, or interventions to improve treatment programmes that did not consist of improved regimens. We identified three studies that assessed the costs or cost-effectiveness of novel treatment regimens; all these studies focused on shorter-duration regimens to treat rifampin-susceptible tuberculosis. Two studies found that shorter regimens with otherwise similar characteristics to the standard of care would yield substantial savings, whereas one study found that a less efficacious, higher-cost, shorter-duration regimen would not be cost-effective in South Africa but could improve treatment outcomes.

**Added value of this study**

This study adds to the existing literature by comparing updated Target Regimen Profiles against improved standards of care (namely the bedaquiline, pretomanid, linezolid, and moxifloxacin regimen for treating rifampin-resistant tuberculosis). Reflecting these improvements, updated targets are increasingly forward-looking and include characteristics not previously analysed, such as regimen forgiveness. Importantly, this study quantifies the cost implications of improving upon individual regimen characteristics beyond duration, helping drug and regimen developers prioritise several potential advances which might present trade-offs.

**Implications of all the available evidence**

Improved regimens to treat tuberculosis could yield substantial cost savings, achieving net cost-neutrality even with higher drug costs. Our results suggest that reductions in regimen duration remain an important contributor to tuberculosis treatment savings, even against improved standards of care and when considering further advances in other regimen characteristics. As characteristics such as regimen efficacy continue to improve, the importance of adherence to treatment and its relationship to the probability of cure increases.

The WHO's Target Regimen Profiles (TRPs), first published in 2016, aim to align regimen development efforts with the needs of people with tuberculosis, care providers, and policymakers by establishing a series of targets, such as reductions in duration and improvements in efficacy, for novel rifampin-susceptible and rifampin-resistant regimens to meet.^16^ In light of the recent advances in treatment regimens, more ambitious targets for future tuberculosis therapeutics are possible and WHO published revised TRPs in 2023.^17^

Moving forward, as novel regimens that meet these targets are developed and evaluated, high drug costs could impede widespread use, despite advantages over existing regimens. Therefore, it is essential for participants in the regimen-development process to be aware of viable price ranges for improved regimens. The full costs of treatment include not only drugs but also other components of treatment, such as outpatient visits and safety-monitoring tests, which often exceed drug costs. A novel regimen with higher drug costs than the current standard-of-care regimen might therefore still yield net savings to both patients and the health system if it reduces the costs of other aspects of treatment. In the longer term, improved regimens could generate additional savings by reducing retreatments and secondary cases, thereby averting future treatment costs. Even if those regimens are not ultimately cost saving, additional costs might still be justified by better health outcomes.

To inform these considerations and the WHO 2023 TRPs, we did a modelling analysis to estimate the price thresholds below which a range of hypothetical novel tuberculosis treatment regimens would yield overall treatment costs no greater than current standards of care (ie, be cost neutral) or offer sufficient value to justify their additional cost (ie, be cost effective). Our analysis was done for three countries with high tuberculosis burden that represented different income levels and geographical regions, comprising India, the Philippines, and South Africa.

## Methods

### Overview

We modelled price thresholds for novel regimens meeting the WHO 2023 TRPs for treating rifampin-susceptible and rifampin-resistant tuberculosis. These thresholds were estimated by comparing the costs and health effects of novel regimens to current standards of care. Novel regimens were hypothetical and based on the TRP targets; they did not correspond to existing regimens. Standards of care were the 6-month isoniazid, rifampin, pyrazinamide, and ethambutol (6HRZE) regimen for rifampin-susceptible tuberculosis and the 6-month BPaLM regimen for rifampin-resistant tuberculosis (including pre-extensively drug-resistant tuberculosis).

Cost-neutral prices were those at which standard-of-care (either rifampin-susceptible or rifampin-resistant) treatment costs were expected to equal costs with a novel (rifampin-susceptible or rifampin-resistant) regimen. Cost-effective prices were those at which the novel regimen increased costs compared with standards of care, but incremental costs per disability-adjusted life-year (DALY) averted equalled country-specific willingness-to-pay thresholds.^18^ We estimated three prices for each novel regimen: short-term cost-neutral prices that considered only savings accrued during the treatment course of a patient, such as from reduced monitoring or shorter duration; medium-term cost-neutral prices that additionally considered savings from averted retreatments and secondary cases over 5 years; and cost-effective prices, measured over a lifetime horizon with costs and DALYs discounted 3% annually.^19^

Analyses were done from both health system (considering medical costs only) and societal (primary analysis; considering medical costs and non-medical patient-borne costs) perspectives.^19^ Three countries (India, the Philippines, and South Africa) were modelled to represent variation in income levels, world regions, and tuberculosis epidemics among countries that account for a large share of the global tuberculosis burden and had data available to parameterise the model.

### Regimen attributes

We analysed hypothetical novel regimens that improved upon standards of care across one or more attributes: efficacy, duration, safety, ease of adherence, and forgiveness (Table 1, Table 2). Efficacy was defined as the proportion of individuals whose tuberculosis would be durably cured (ie, microbiological cure without future relapse) if they completed the full regimen duration with adequate adherence. Ease of adherence encompassed several regimen characteristics that influence adherence (ie, percentage of doses taken while remaining on treatment), including tolerability, pill burden, and dosing frequency. Forgiveness described the effect of non-adherence on outcomes; with more forgiving regimens, more patients with low adherence would still be cured. Finally, safety influenced adverse event frequency and toxicity monitoring. WHO's 2023 TRPs established minimal and optimal targets for several regimen characteristics; we modelled regimens meeting the optimal TRP targets across all five attributes (TRP-optimal), regimens meeting only the minimal TRP targets for all attributes (TRP-minimal), and regimens with a combination of optimal, minimal, and standard-of-care-equivalent attributes.

**Table 1:** Influence of novel regimen attributes on costs

	**Definition in the analysis**	**Effects of improvements in the analysis**
Efficacy	Percentage durably cured (ie, without relapse), among patients who were adequately adherent and who completed the full regimen duration	More patients are cured, averting morbidity, mortality, retreatment costs, and secondary cases
Duration	Intended duration (months)	A higher proportion of patients complete enough treatment to be durably cured; fewer outpatient visits and laboratory tests are required; treatment support costs are lower; patient out-of-pocket and indirect time costs are lower; and fewer cumulative adverse events occur
Ease of adherence	Percentage of doses taken (while a patient remains on treatment)	More patients take enough doses to be durably cured
Forgiveness	Percentage of doses that can be missed without loss of efficacy	More patients with imperfect adherence are durably cured
Safety	Adverse event and toxicity profile of the regimen*	Less monthly safety monitoring is required, reducing laboratory test costs; monthly incidence of adverse events is lower

* In the analysis, safety influences the percentage of patients that had adverse events each month as well as toxicity monitoring requirements.

**Table 2:** Model parameters

		**Standard of care**	**TRP-minimal**	**TRP-optimal**	**Sources and notes**
**Rifampin-susceptible tuberculosis regimen attributes**					
Efficacy		95% (94–97)^20^	95% (94–97)	99%	TRP-minimal efficacy was as effective as standard of care and TRP-optimal efficacy was better than that of standard of care
Duration		6 months	3·5 months	2 months	The standard of care was assumed; TRP-minimal duration was the median of the minimal range in the TRPs (3–4 months); and TRP-optimal duration was the optimal value in the TRPs
Ease of adherence (percentage of patients by adherence category)		..	..	..	TRP-minimal ease of adherence was the same as standard of care and TRP-optimal ease of adherence was consistent with that of a long-acting injectable formulation
	<70%	38% (28–48)^21^, ^22^, ^23^	38% (28–48)	0%	..
	70–85%	10% (4–16)^21^, ^22^, ^23^	10% (4–16)	0%	..
	85–90%	21% (14–30)^21^, ^22^, ^23^	21% (14–30)	0%	..
	≥90%	31% (22–40)^21^, ^22^, ^23^	31% (22–40)	100%	..
Forgiveness (percentage of doses that can be missed while still achieving full efficacy)		10% (5–17%)^24^	15% (7–25%)	30% (15–50%)	TRP-minimal had 1·5 times better forgiveness than standard of care and TRP-optimal had three times better forgiveness than standard of care
Drug prices for a full course of 6HRZE, US dollars*		$46^25^	..	..	..
**Rifampin-resistant tuberculosis regimen attributes**					
Efficacy		89% (83–94)^6^, ^7^, ^9^	89% (83–94)	97%	TRP-minimal efficacy was as effective as standard of care, and TRP-optimal efficacy was the median of the TRP-minimal and TRP-optimal efficacy values for rifampin-susceptible tuberculosis
Duration		6 months	6 months	2 months	The standard of care was assumed; TRP-minimal and TRP-optimal durations matched the minimal and optimal duration values in the TRPs
Ease of adherence (percentage of patients by adherence category)		..	..	..	Standard of care was the minimum adherence across the three studies used for rifampin-susceptible tuberculosis standard of care,^23^ given the the scarcity of evidence on adherence under BPaLM and its lower tolerability (side-effects); TRP-minimal ease of adherence was the same as TRP-minimal ease of adherence for rifampin-susceptible tuberculosis; and that of TRP-optimal was consistent with a long-acting formulation
	<70%	40% (31–49%)	38% (28–48%)	0%	..
	70–85%	24% (18–30%)	10% (4–16%)	0%	..
	85–90%	11% (4–20%)	21% (14–30%)	0%	..
	≥90%	25% (16–34%)	31% (22–40%)	100%	..
Forgiveness (% doses that can be missed while still achieving full efficacy)		15% (7–25%)	15% (7–25%)	30% (15–50%)	Standard of care^24^ was adjusted for pharmacokinetic evidence (eg, bedaquiline and pretomanid have longer half-lives than all the component drugs in HRZE);^26^ TRP-minimal forgiveness was the same as standard of care; TRP-optimal forgiveness was the same as rifampin-susceptible TRP-optimal forgiveness
Drug prices, BpaLM, full course		$432^25^	..	..	..
Drug prices, BPaL, full course		$404^25^	..	..	..

BPaLM=bedaquiline, pretomanid, linezolid, and moxifloxacin. TRP=Target Regimen Profile. 6HRZE=6 months of isoniazid, rifampin, pyrazinamide, and ethambutol.

* Drug prices under the rifampin-susceptible standard of care were $85 for patients with identified isoniazid resistance due to substitution of levofloxacin.^25^

### Model of patient outcomes

Our decision-analytical model followed cohorts of adults with either rifampin-susceptible or rifampin-resistant tuberculosis (patients), initiating treatment with a regimen appropriate to their rifampin resistance (appendix pp 3–5). Patients with undetected rifampin resistance, whose treatment outcomes were expected to be poor for any rifamycin-containing regimen, were not included in the analysis. For resistance to other component drugs (isoniazid and fluoroquinolones), the prevalence and detection of resistance, and corresponding effects on standard-of-care outcomes and costs, were based on country-specific evidence (table 2; appendix p 6).

The proportion of patients cured was modelled as a function of efficacy, duration, ease of adherence, and forgiveness. Patients were assigned to one of four adherence categories (<70%, 70–85%, 85–90%, or ≥90% of mean doses taken per week). These categories were selected on the basis of data from adherence-monitoring studies,^21^, ^22^, ^23^ which were used to parameterise the standard-of-care adherence distribution. The effects of regimen forgiveness were modelled as a step function, on the basis of an individual data synthesis suggesting an 18% lower probability of cure with 6HRZE for patients with adherence lower than 90%.^24^ For patients with non-adherence higher than the forgiveness threshold of a regimen (10% for 6HRZE, 15% for BPaLM, and 30% for more forgiving novel regimens), efficacy was reduced by the same 18% amount.

Patients had a 0·3% weekly probability of premature discontinuation^27^ that was fixed over time^28^ and across regimens; the cumulative risk of discontinuation was thus higher for longer-duration regimens. The effect of premature discontinuation was modelled for all regimens as a functional relationship between the proportion of the intended duration completed and the probability of cure (appendix p 7). Patients also faced a weekly, regimen-dependent probability of experiencing non-treatment-discontinuing adverse events that reduced quality of life (resulting in DALY accrual).

### Medium-term health outcomes

We estimated mortality, retreatments, and secondary cases from the modelled proportion of patients not experiencing durable cure under each regimen (including immediate microbiological failures and future relapses). We assumed country-specific tuberculosis and non-tuberculosis mortality for these patients; those who did not die were assumed to be retreated after a mean of 1·65 years and to generate a mean of one secondary case of tuberculosis (appendix p 4). Secondary cases were treated according to country-specific case-detection ratios,^1^ with the same mean health outcomes as primary cases; they could also die before being linked to care. Further details, including disability weights, are available in the appendix (pp 6, 8–9).

### Cost estimates

For each standard-of-care and novel regimen, we did an ingredients-based costing analysis (appendix p 10). Categories of costed inputs included drugs, outpatient visits, laboratory tests and diagnostics, hospitalisation, patient support, and adverse-event management. Patient-borne out-of-pocket non-medical and indirect time costs were included in the societal perspective analysis only. We assumed that each country-specific unit cost (except drugs) would be fixed across regimens, but the quantities of each input would vary by regimen (appendix pp 12–16). In the medium-term cost-neutrality and cost-effectiveness analyses, treatment of recurrent and secondary cases incurred these same mean costs.

For novel regimens, several quantities scaled with patient time on treatment, including outpatient visits, treatment support, laboratory tests, adverse-event incidence, and patient costs. The number and type of laboratory tests for drug toxicity scaled with regimen safety. All costs were expressed in 2021 US dollars.

### Additional details

Uncertainty in all model parameters was incorporated via probabilistic sensitivity analysis by running the model with 10 000 parameter set samples drawn from literature-based uncertainty distributions and calculating means and 95% uncertainty intervals (UIs; 2·5th and 97·5th percentiles) across the 10 000 resulting modelled output samples. Uncertainty in willingness to pay was assessed by evaluating cost-effective prices under a range of thresholds for each sample.

Our analysis conforms to Consolidated Health Economic Evaluation Reporting Standards (appendix p 17).^29^ Updates made to this analysis since earlier iterations that informed the 2023 TRPs are described in the appendix (p 24). All analyses were done using R version 4.2.2 and model code is available on Github. Given that no individual-level data were used in this analysis, this study did not meet the definition of human patient research at Johns Hopkins University and thus ethical approval was not required.

### Role of the funding source

WHO had no role in the study design, data collection, data analysis, writing of this report, or the decision to submit for publication.

## Results

**Figure 1:**
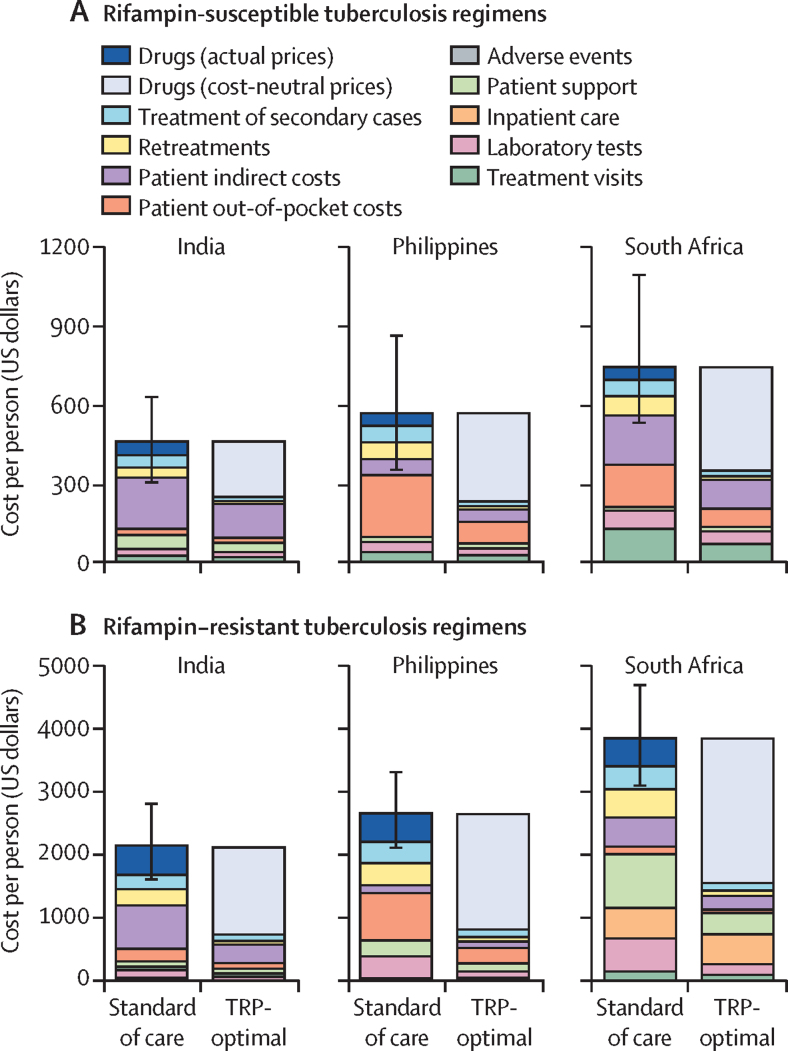
Medium-term societal costs of standard of care and improved tuberculosis treatment regimens Estimated mean medium-term costs per patient with rifampin-susceptible tuberculosis treated with the standard of care (6 months of isoniazid, rifampin, pyrazinamide, and ethambutol for most patients) and with regimens meeting all optimal targets in the 2023 TRPs (A). Estimated mean costs per patient with rifampin-resistant tuberculosis treated with the standard of care (6 months of bedaquiline, pretomanid, linezolid, and moxifloxacin for most patients) and TRP-optimal regimens (B). TRP-optimal regimen costs include the costs of drugs when priced at their estimated medium-term cost-neutral prices (lightest blue), whereas standard-of-care regimen costs include the cost of drugs at their current prices (dark blue). The difference in the heights of the non-drug (non-lightest blue and non-dark blue) bars within each panel thus represent medium-term non-drug cost savings from an optimla TRP regimen. Shading indicates mean costs attributed to different components of treatment and error bars indicate uncertainty intervals (2·5th and 97·5th percentiles across 10 000 parameter set samples) for total cost estimates under the standard of care. The heights of the lightest blue bars (drug costs at cost-neutral prices) for the TRP-optimal regimens differ slightly from the thresholds shown in table 3 (cost-neutral prices) because of wastage and incomplete person-time on treatment. Note the difference in y-axis scales between panels A and B. TRP=Target Regimen Profile.

Under the rifampin-susceptible-tuberculosis standard of care (6HRZE for most patients), estimated short-term mean costs per treatment course under the societal perspective, which included both patient and health system costs, were US$380 (95% UI 270–520) in India, $450 (290–690) in the Philippines, and $610 (450–910) in South Africa (figure 1). Patient-borne costs accounted for 61% (46–73%) of total costs in India, 69% (53–81%) in the Philippines, and 58% (45–73%) in South Africa. An estimated 17% (95% UI 9–30) of patients were not durably cured, resulting in additional costs of $70 (30–135) in India, $110 (50–220) in the Philippines, and $120 (50–240) in South Africa per case for retreatment and treatment of secondary cases. Resulting medium-term costs per person were $450 (95% UI 310–630) in India, $560 (350–860) in the Philippines, and $730 (530–1090) in South Africa.

Improved rifampin-susceptible-tuberculosis regimens yielded substantial savings, allowing these hypothetical novel regimens to reach net-cost neutrality even if priced higher than the standard of care (table 3; figure 1). A TRP-optimal rifampin-susceptible-tuberculosis regimen (meeting all optimal targets in the TRPs) was estimated to be cost-neutral in the short-term at prices of $140 (95% UI 90–210) per full course in India, $230 (130–380) in the Philippines, and $280 (180–460) in South Africa, compared with $46 for 6HRZE. The greatest short-term savings were from lower patient-borne costs. Durable cures increased by 20% (95% UI 9–41) compared with the standard of care, and considering resulting savings increased cost-neutral prices in the medium term by $50–100 for the three countries ($190, 95% UI 130–290 in India, $320, 180–530 in the Philippines, and $380, 240–620 in South Africa). Under a health systems perspective that considered only medical costs, cost-neutral prices were 46–50% lower in India (a reduction of 46% in the short term and 50% in the medium term), 70% lower in the Philippines, and 57% lower in South Africa.

**Table 3:** Cost-neutral and cost-effective price thresholds for TRP-optimal novel rifampin-susceptible and rifampin-resistant tuberculosis treatment regimens under societal and health system perspectives, in US dollars

		**India**	**Philippines**	**South Africa**
**Regimens for rifampin-susceptible tuberculosis**				
Short-term cost-neutral threshold				
	Health system	70 (70–80)	70 (60–80)	120 (100–140)
	Societal	140 (90–210)	230 (130–380)	280 (180–460)
Medium-term cost-neutral threshold				
	Health system	100 (80–120)	100 (80–120)	160 (130–210)
	Societal	190 (130–290)	320 (180–530)	380 (240–620)
Cost-effective threshold				
	Health system	950 (510–1740)	1570 (800–2900)	6440 (3170–12 130)
	Societal	1050 (580–1870)	1800 (970–3200)	6660 (3370–12 440)
**Regimens for rifampin-resistant tuberculosis**				
Short-term cost-neutral threshold				
	Health system	490 (470–510)	730 (670–790)	1250 (1030–1520)
	Societal	930 (720–1230)	1180 (980–1430)	1480 (1230–1780)
Medium-term cost-neutral threshold				
	Health system	610 (550–700)	960 (820–1150)	1680 (1320–2140)
	Societal	1200 (910–1630)	1610 (1260–2070)	2010 (1590–2560)
Cost-effective threshold				
	Health system	1580 (1090–2300)	2700 (1800–3980)	8800 (5350–13 820)
	Societal	2190 (1550–3060)	3370 (2340–4830)	9140 (5640–14 230)

Mean estimated cost-neutral and cost-effective prices per full treatment course, with 95% uncertainty intervals. Regimens were modelled as having efficacy, duration, ease of adherence, forgiveness, and safety consistent with the optimal targets in the WHO 2023 TRPs. Price thresholds are rounded to the nearest ten. TRP=Target Regimen Profile.

Given that better regimens also improve treatment outcomes, novel regimens could be priced substantially higher than the cost-neutral prices and still be cost-effective compared with standards of care. The cost-effective prices estimated for a TRP-optimal rifampin-susceptible-tuberculosis regimen were $1050 (95% UI 580–1870) in India, $1800 (970–3200) in the Philippines, and $6660 (3370–12 440) in South Africa.

For rifampin-resistant tuberculosis, short-term standard-of-care costs (BPalM) were higher than for rifampin-susceptible tuberculosis, at $1700 (95% UI 1310–2250) per patient in India, $2000 (1650–2420) in the Philippines, and $3050 (2550–3630) in South Africa. Patient-borne costs comprised a smaller share of the total. Outcomes were also worse for rifampin-resistant tuberculosis (22%, 95% UI 13–33, not durably cured); adding retreatment and secondary-case costs thus increased total costs more for the rifampin-resistant standard of care than for the rifampin-susceptible standard of care (23–30% increase over short-term costs).

A TRP-optimal rifampin-resistant-tuberculosis regimen was estimated to be cost-neutral in the short term at prices of $930 (95% UI 720–1230) in India, $1180 (980–1430) in the Philippines, and $1480 (1230–1780) in South Africa (*vs* $432 per full course of BPaLM).Medium-term cost-neutral prices were $1200 (910–1630) in India (a 30% increase compared with the short-term cost-neutral price), $1610 (1260–2070) in the Philippines (a 37% increase), and $2010 (1590–2560) in South Africa (a 36% increase).

Similar to rifampin-susceptible tuberculosis, the greatest savings were from reductions in patient costs and averted retreatments and secondary cases (mean 24%, 95% UI 11–44 more patients durably cured). Decreases in laboratory and patient-support costs also made up a substantial share of savings. The importance of considering patient-borne costs varied by country; in India and the Philippines, health-system cost-neutral thresholds were 39–49% lower than under a societal perspective. In South Africa, where support vouchers (paid by the health system) supplanted some costs that would otherwise be borne by patients, health system thresholds were only 15–16% lower than under a societal perspective. Cost-effective prices were much higher, at $2190 (95% UI 1550–3060) in India, $3370 (2340–4830) in the Philippines, and $9140 (5640–14 230) in South Africa.

**Figure 2:**
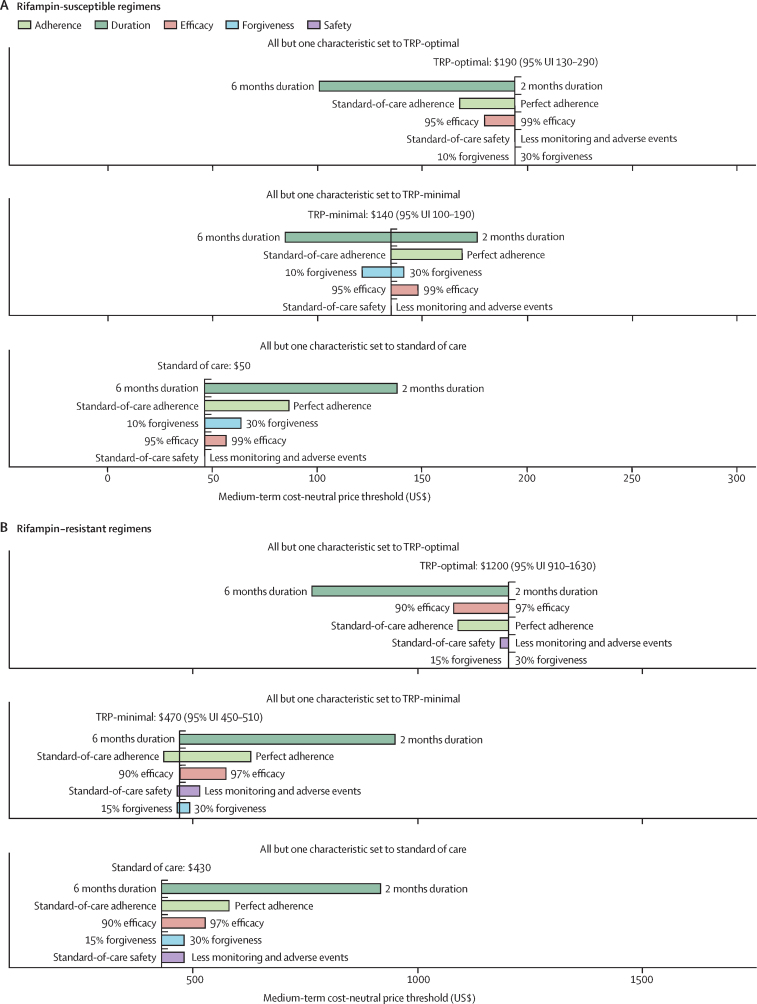
Influence of five novel tuberculosis-treatment regimen attributes on medium-term cost-neutral price thresholds in India Medium-term cost-neutral price thresholds for novel rifampin-susceptible (A) and rifampin-resistant (B) regimens in India under the societal perspective. Coloured bars show the variation in the price threshold when a single characteristic is varied from its standard-of-care value to its optimal TRP value. The values that the non-varying characteristics take on differ between the three sections of each panel; the bottom sections of both panels (all but one characteristic set to standard of care) show thresholds when all non-varying characteristics are fixed at their standard-of-care values (vertical dashed line), the middle sections of each panel (all but one characteristic set to minimal TRP) show results when all non-varying characteristics are fixed at their minimal target values from the TRPs (table 2), and the top sections of each panel (all but one characteristic set to optimal TRP) show results when all non-varying characteristics are fixed at their optimal values from the TRPs. Colours indicate which characteristic is being varied, and text labels indicate the values of each characteristic (left of the bars, standard-of-care values for each characteristic; right of the bars, TRP-optimal values for each characteristic). Bars are ordered vertically by the effect each characteristic has on the threshold (the vertical distance between each bar is equal and not meaningful). TRP=Target Regimen Profile. UI=uncertainty interval.

In analyses which varied one attribute at a time (from the standard-of-care value to the TRP-optimal value) while holding all other attributes fixed (at standard-of-care, TRP-minimal, or TRP-optimal values), shortening regimen duration was estimated to yield the most savings for both rifampin-susceptible and rifampin-resistant tuberculosis treatment, thus increasing cost-neutral prices by the greatest amount (figure 2). For rifampin-susceptible tuberculosis, shortening from 6 months to 2 months increased medium-term cost-neutral prices by approximately $90–240, depending on the comparator and country. For rifampin-resistant tuberculosis, equivalent duration reductions increased cost-neutral prices by $440–490 for India, $630–750 for the Philippines, and $780–1000 for South Africa.

Ease of adherence was the next most influential driver of medium-term savings. Increasing adherence to 100% for all patients (ie, such as might be achieved through a long-acting injectable) led to cost-neutral prices that were around $30–70 (rifampin-susceptible tuberculosis) and $110–290 (rifampin-resistant tuberculosis) greater than prices of otherwise-equivalent regimens with standard-of-care adherence. Interactions between forgiveness and adherence yielded interrelated effects on costs. By contrast to the short-term cost-saving benefits of shortened durations, the economic benefits of improving characteristics that primarily affected treatment outcomes (adherence, forgiveness, and efficacy) were most apparent in the medium-term and cost-effectiveness analyses. Results were qualitatively similar across countries (appendix pp 27–32).

Safety had less of an effect on costs but yielded some laboratory and adverse-event management savings. Improved safety affected rifampin-resistant-tuberculosis costs more (cost-neutral prices around $20–270 higher than otherwise equivalent regimens with standard of care safety) given the poorer safety profile of BPaLM compared with 6HRZE. Safety improvements led to meaningful reductions in the incidence of adverse events, resulting in 11 (95% UI 4–23) DALYs averted per every 100 people treated in India, 11 (4–24) in the Philippines, and ten (3–21) in South Africa

For both rifampin-susceptible and rifampin-resistant tuberculosis, cost-effective prices increased with willingness to pay, which varied by country (appendix pp 35–40). Given that these thresholds represent willingness to pay for averted DALYs, prices increased more when attributes with the greatest effect on treatment outcomes were improved (adherence, efficacy, and forgiveness).

## Discussion

We found that regimens meeting the optimal targets set in the 2023 WHO TRPs could yield substantial savings compared with current standards of care. Such regimens could be cost neutral in the medium term even at drug costs four-to-eight times higher than current rifampin-susceptible regimen costs (for novel rifampin-susceptible regimens) and three-to-five times higher than current rifampin-resistant regimen costs (for novel rifampin-resistant regimens). Savings accrued primarily in the form of reduced patient costs (driven mostly by shortened durations) and, for rifampin-resistant tuberculosis, reductions in the number of retreatments and secondary cases (driven by improvements in adherence, efficacy, and forgiveness). Cost-neutral and cost-effective prices varied by setting, on the basis of country-specific unit costs, treatment protocols, non-medical patient-borne costs, and willingness to pay. Of the five attributes analysed, shortened duration yielded the most savings for both rifampin-susceptible-tuberculosis and rifampin-resistant-tuberculosis regimens across all three modelled countries, but had little effect on cures. Improved ease of adherence generally had the second-greatest effect on costs and the greatest effect on the percentage of patients durably cured.

These results suggest that, from a cost perspective, duration and ease of adherence are particularly important attributes to consider in the development and evaluation of novel tuberculosis drugs and regimens. Importantly, this analysis is not intended to justify novel drug prices that are higher than the cost of goods, but rather to demonstrate that costlier drugs might be economically feasible to introduce in high-burden settings if they can meaningfully outperform existing standards of care.

Strengths of this analysis include consideration of a range of regimen improvements in three large high-burden countries, allowing for generalisability to future novel regimens across many low-income and middle-income countries with high tuberculosis burdens. In very-low-resource settings with insufficiently strengthened health systems, adherence might be lower than that modelled here; if novel regimens can still improve adherence to the extent we assumed, cost-neutral and cost-effective prices are expected to be higher. However, these price threshold increases might be offset by lower willingness (or ability) to pay.

Tuberculosis disproportionately affects people living in poverty,^30^ and these results incorporate the substantial economic burden so often placed on people with tuberculosis. Half of tuberculosis-affected households face catastrophic costs (costs >20% of annual income)^1^ and many are forced to engage in financially damaging coping strategies.^31^ Treatment improvements could support global efforts to eliminate these financial hardships.^32^, ^33^ We found that TRP-optimal regimens could reduce patient-borne non-medical costs by 29–56%, primarily through reduced duration. Shortened regimens could thus mitigate against poverty resulting from tuberculosis disease and promote equity. Because tuberculosis drugs are available free of charge through the public sector in most countries, these equity benefits are likely to accrue provided a novel regimen is priced low enough for a health system to adopt it and access is equitable. Given that patients rarely pay out of pocket for tuberculosis drugs, we did not estimate drug-price thresholds from a patient-only perspective.

Previous literature has similarly found that duration-shortening regimens could yield substantial savings^34^ if such treatment shortening does not compromise efficacy.^35^ This study adds to that literature by incorporating improving standards of care and correspondingly forward-looking target-regimen profiles. Importantly, we explored the effects of individual regimen characteristics to help prioritise those likely to have the greatest influence on achieving savings and cost-effectiveness.

Because these results are based on total spending across a variety of cost categories, they should not be interpreted as describing budgetary impact. Short-term health system estimates might be the most representative of cost neutrality from an annual country-budgeting standpoint. However, even within single-year health budgets, shifting costs across different spending categories could be difficult, particularly if some costs are more typically borne by external partners. As such, scenarios that are cost-saving to the health system or society as a whole might still increase tuberculosis programme budgets. Furthermore, some projected savings might not be fully realised. For example, a reduction in the number of tuberculosis-focused clinic visits might not directly translate into commensurate cost savings unless staffing is downsized, although it could free up staff time for other tasks. On the other hand, these results do not incorporate the potential to reduce tuberculosis costs more broadly through reductions in tuberculosis incidence.^36^ Finally, current standards of care might still present substantial cost barriers to many tuberculosis programmes, and thus regimens that achieve net cost-neutrality might still be unaffordable in many settings.

Other limitations of this analysis include the uncertain costs and risks of drug-resistance acquisition during treatment (which were not considered),^37^ the uncertain costs of novel drug-susceptibility testing (which might differ from current unit costs), scarce evidence on the forgiveness of, and adherence to, standard-of-care regimens (forgiveness was estimated from one study on the risk of composite unfavourable outcomes among patients treated with 6HRZE),^24^ the potentially limited generalisability to children and other physiologically distinct populations, and the potential for reductions in standard-of-care costs to drive down cost-neutral thresholds. Meaningful reductions in cost are unlikely for 6HRZE, but BPaLM prices fell substantially in 2023^25^ and could decline further with generic licensing.^38^ Although rollout of the 4-month isoniazid, rifapentine, moxifloxacin, and pyrazinamide regimen for rifampin-susceptible tuberculosis has been limited in high-burden settings,^39^ further declines in the price of rifapentine^25^ could encourage its uptake as a new standard of care, thus reducing savings from even shorter (<4 month) rifampin-susceptible-tuberculosis regimens.

Finally, considering only costs and DALYs does not fully capture the mechanisms by which regimen advances might improve the quality of life of people on tuberculosis treatment. Future analysis of tuberculosis treatment regimens would benefit from more research on the influence of characteristics such as side-effect profiles, pill burden, and duration on both adherence and the broader experiences of people with tuberculosis. Although not included in the TRPs, the potential for treatment to reduce the incidence and severity of post-tuberculosis disability is another important consideration.^40^

This analysis of the economic implications of improved rifampin-susceptible and rifampin-resistant tuberculosis therapeutics found that novel regimens, particularly shorter regimens or regimens that are easier to adhere to, could yield substantial non-drug-related savings while also increasing cures and presumably improving patient experiences. Taken together, these results suggest that higher-cost novel tuberculosis treatment regimens with characteristics leading to meaningful improvements over current options could be economically viable in a range of settings.

## Data sharing

All data used in this study are from publicly available sources that are listed in table 2 and in the appendix (pp 6–9, 12–16) and are cited in the references. All data-sharing enquiries should be addressed to TSR.

## Declaration of interests

TSR, CL, DWD, and EAK report funding from WHO. TSR and EAK report funding from the Bill & Melinda Gates Foundation. All other authors declare no competing interests.
