# Supplementary material for: Economic implications of novel regimens for tuberculosis treatment in three high-burden countries: a modelling analysis
Source: Lancet Glob Health. 2024 May 16;12(6):e995–e1004. doi: 10.1016/S2214-109X(24)00088-3 (PMC11126367; doi:10.1016/S2214-109X(24)00088-3)
Supplement: Supplementary appendix [file mmc1.pdf]

THE LANCET
Global Health

Supplementary appendix

This appendix formed part of the original submission and has been peer reviewed. We post it as supplied by the authors.

Supplement to: Ryckman TS, Schumacher SG, Lienhardt C, et al. Economic implications of novel regimens for tuberculosis treatment in three high-burden countries: a modelling analysis. *Lancet Glob Health* 2024; **12:** e995–1004.

Supplementary Appendix for "Economic implications of novel regimens for TB treatment in three high-burden countries: a modeling analysis"

Table of Contents

Supplementary Methods 3
Figure S1: TB treatment model structure 3
Text S1: Additional Patient Cures Modeling Details 3
Table S1: Country-Specific Model Parameters 6
Figure S2: Modeled relationship between loss-to-follow-up, duration, and probability of cure 7
Table S2: Disability weights 8
Figure S3: Estimated serial interval distribution 9
Text S2: Additional Cost Analysis Details 10
Table S3: Country-Specific Health System Unit Costs 12
Table S4: Country-Specific Patient-Borne Unit Costs 14
Table S5: Quantities of treatment inputs and events in the cost analysis 15
Table S6: CHEERS checklist for economic evaluation studies 17
Supplementary Methods References 19
Differences Compared to Modeling in the WHO TRP Document 24
Text S3: Summary of methodological differences 24
Table S7: Comparison of price threshold estimates 25
Supplementary Results 26
Table S8: Impact of RS-TB regimen improvements on short-term cost-neutral price thresholds 27
Table S9: Impact of RR-TB regimen improvements on short-term cost-neutral price thresholds 28
Table S10: Impact of RS-TB regimen improvements on medium-term cost-neutral price thresholds ... 29
Table S11: Impact of RR-TB regimen improvements on medium-term cost-neutral price thresholds ... 30
Table S12: Impact of RS-TB regimen improvements on cost-effective price thresholds 31
Table S13: Impact of RR-TB regimen improvements on cost-effective price thresholds 32
Table S14: Impact of RS-TB regimen improvements on DALYs and percent of patients durably cured 33
Table S15: Impact of RR-TB regimen improvements on DALYs and percent of patients durably cured 34
Figure S4: Cost-effective prices with variation in willingness-to-pay: India, societal perspective 35
Figure S5: Cost-effective prices with variation in willingness-to-pay: India, health systems perspective 36
Figure S6: Cost-effective prices with variation in willingness-to-pay: South Africa, societal perspective 37

Figure S7: Cost-effective prices with variation in willingness-to-pay: South Africa, health systems perspective 38
Figure S8: Cost-effective prices with variation in willingness-to-pay: Philippines, societal perspective 39
Figure S9: Cost-effective prices with variation in willingness-to-pay: Philippines, health systems perspective 40

Supplementary Methods

Figure S1: TB treatment model structure

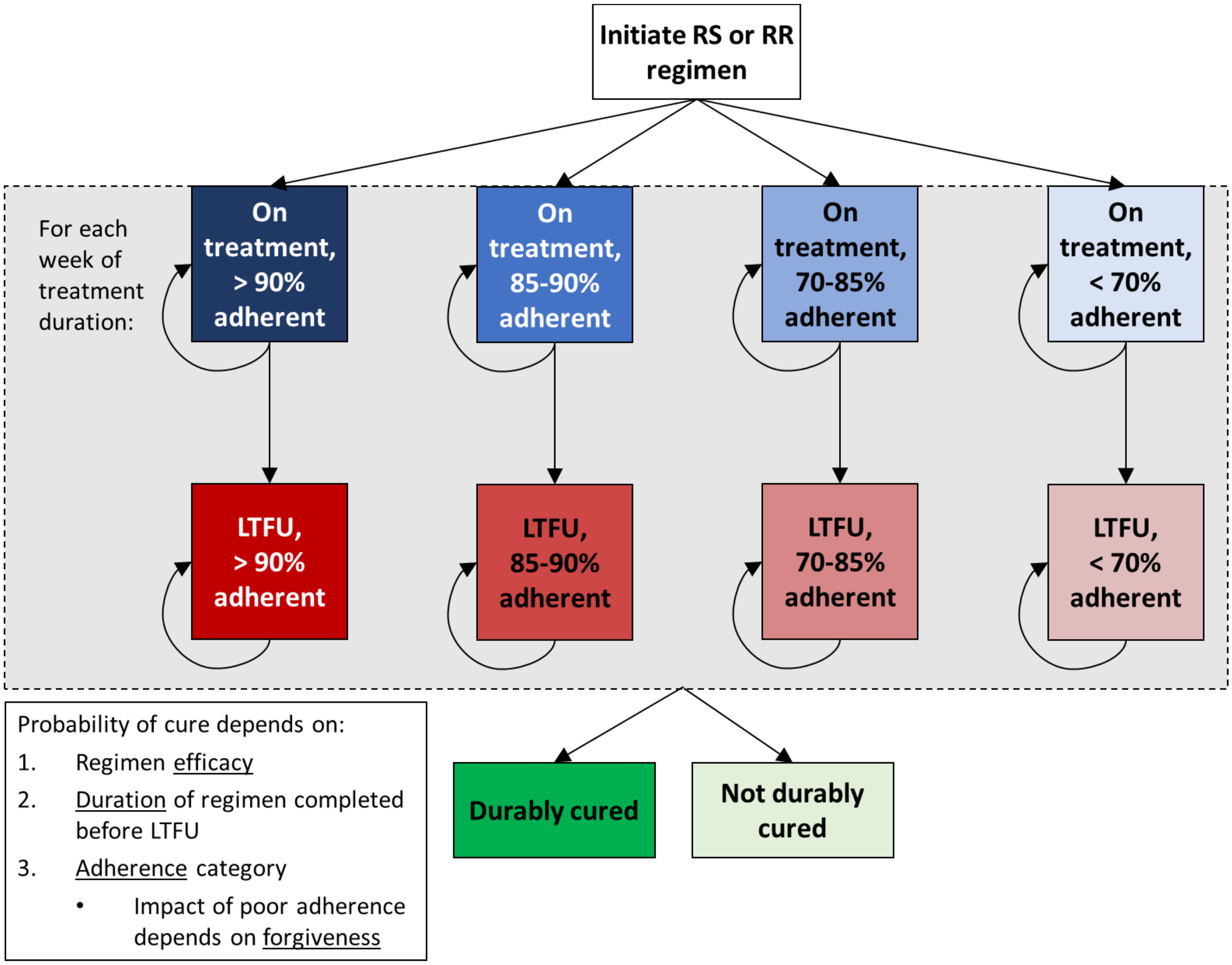

After a patient initiates a rifampin-susceptible (RS) or rifampin-resistant (RR) regimen (either the standard of care or a novel regimen), their probability of cure depends on average weekly doses taken (adherence) while on treatment; loss-to-follow-up (LTFU) during treatment (and when that LTFU occurred), which is modeled with a weekly timestep; regimen efficacy; and regimen forgiveness. In the figure, boxes indicate patient states while arrows indicate transitions between states. See Text S1 for additional information.

Text S1: Additional Patient Cures Modeling Details

Main Model

Among rifampin-susceptible and rifampin patients who are eligible for (based on rifampin-resistance) and initiate a regimen, we estimated the proportion of patients durably cured as a function of regimen efficacy, duration, ease of adherence, and forgiveness. Regimen **efficacy** represented the proportion cured among patients who are 100% adherent and take the full course of treatment, and is based on outcomes from clinical trial data. The probability of cure was then reduced from this maximal amount among those who are less than fully adherent ($adj\_adherence$) or complete less than the intended duration ($adj\_duration$).

$$[1] \qquad p\_cure_i = (efficacy_i)(adj\_adherence_i)(adj\_duration_i)$$

Specifically, with regard to adherence, we modeled the proportion of patients who are less than fully adherent as depending on the **ease of adherence**, and we modeled the extent to which that nonadherence reduced the probability of cure (below the regimen's maximal efficacy) as depending on the regimen's **forgiveness**. Adherence is defined as the percent of doses a patient takes while they remain in care (not lost to follow up). We divided patients into two adherence categories: adequate adherence ($p_{adequate}$) and reduced adherence ($p_{reduced}$), where the probability of cure is only reduced for those in the reduced adherence group. The threshold determining adequate vs. reduced adherence is based on forgiveness. Specifically, forgiveness is defined as the percent of doses that can be missed at which patients are still expected to achieve full regimen efficacy. The relative efficacy achieved by poorly adherent patients – 77% - is based on evidence from the 6-month HRZE regimen (1) and does not vary across regimens. For example, our model would estimate that under the standard of care for RS-TB (forgiveness = 10%), 95% of the 31% of patients that took ≥ 90% of prescribed doses each week would be cured cured, while only 77% of the other 69% that took < 90% of prescribed doses would be cured. For any given threshold, the proportion of patients with adequate adherence increases with better ease of adherence.

$$[2] \qquad adj\_adherence_i = \frac{1}{N}\sum_{n=1}^{N} 1\{adherence \geq threshold_{forgiveness;i}\}_{ease\_adherence;\, i} + (releff_{reduced\_adherence})(1 - \frac{1}{N}\sum_{n=1}^{N} 1\{adherence < threshold_{forgiveness;i}\}_{ease\_adherence;\, i})$$

Finally, the regimen **duration** determines the probability that a patient completes the full regimen and experiences the associated probability of cure (i.e., the efficacy modified by an adherence-dependent factor). The duration also determines what proportion of efficacy is lost when patients are lost to follow up after a given partial duration, because a given number of treatment weeks will be a greater proportion of the full treatment course (and will thus achieve closer to full efficacy) for a regimen whose full duration is shorter. We model the relationship between percent of regimen completed and percent of efficacy realized as being constant across regimens ($releff_t$; Figure S1). To determine the patients lost to follow up at each weekly time step t, we model a non-regimen-varying loss-to-follow-up risk of 1·1% per month (0·3% per week) over a regimen's intended duration; thus, cumulative loss-to-follow-up is higher for regimens with longer durations.

$$[3] \qquad adj\_duration_i = (1 - 0 \cdot 003)^{duration_i} + \sum_{t=1}^{duration_i - 1} 0 \cdot 003(1 - 0 \cdot 003)^{t-1}\, releff_t$$

Outcomes among patients initiating treatment are thus determined by a product of efficacy, loss-to-follow-up, duration, adherence, and forgiveness, as shown in equation 4.

$$[4] \quad p_{cure;i} = efficacy_i * \left[\frac{1}{N}\sum_{n=1}^{N} 1\{adherence \geq threshold_{forgiveness;i}\}_{ease\_adherence;\, i} + (releff_{reduced\_adherence})(1 - \frac{1}{N}\sum_{n=1}^{N} 1\{adherence < threshold_{forgiveness;i}\}_{ease\_adherence;\, i})\right] * \left[0 \cdot 997^{duration_i} + \sum_{t=1}^{duration_i - 1} 0 \cdot 003(0 \cdot 997)^{t-1} releff_t\right]$$

Post-treatment outcomes

After estimating the proportion of patients durably cured by a regimen, we then projected the impact this would have on retreatments and secondary cases (parameters in Table S1). A portion of failures and relapses (e.g., all patients that were not durably cured), were assumed to die of TB-related causes, based on WHO data on the proportion of treatment non-successes that die and assuming only half of these deaths could be averted by improved regimens that result in more cures (2). All failures and relapses who remained alive immediately after treatment were assumed to eventually die of TB or be retreated; retreatment was assumed to start an average of 1·65 years after the start of the initial treatment course, based on cohort data on the timing of relapse among patients who completed treatment with or without documented cure (3). We additionally assumed that each patient who was not cured (but remained alive after treatment) generated an average of 1 secondary case after their unsuccessful treatment attempt; this incorporated assumptions of an effective reproduction number near 1, and of similar cumulative transmission from recurrent and new TB episodes (4). These secondary cases were assumed to occur a median of 1·9 years after the initial unsuccessful treatment, based on estimates of the TB serial interval and incorporating an additional 6 months average delay from initiation of treatment to recurrent infectiousness (5,6) (see Figure S3 on appendix page 9). Secondary cases faced country-specific case fatality ratios and those that did not die were put on treatment, with timing according to country-specific case detection ratios.

Resistance to rifampin

We only modeled RR-TB patients who initiated an RR-TB regimen; that is, no patients with RR-TB were modeled as initiating a rifamycin-containing (RS-TB) regimen. This assumption was made because we were not modeling pan-TB regimens as defined by WHO's target regimen profiles, and novel RS- and RR-TB regimens are not expected to change the proportion of patients receiving rifampin drug susceptibility testing, the costs of that drug susceptibility testing, or the fact that treatment outcomes are poor for patients with undetected rifampin resistance who receive a rifamycin-containing regimen. Therefore, price thresholds are not expected to be affected by the consideration of people with RR-TB receiving an RS-TB regimen.

Resistance to isoniazid and fluoroquinolones

We assumed that some rifampin-susceptible patients would be resistant to isoniazid and some rifampin-resistant patients would be resistant to fluoroquinolones (i.e., pre-XDR TB). Only a subset of resistant patients were assumed to have their resistance status detected, based on country-specific data on drug susceptibility testing practices (2). Under the standard of care, patients with detected component drug resistance would instead initiate an alternative regimen appropriate for their known drug resistance (6-month levofloxacin rifampin pyrazinamide ethambutol for RS-TB; 6-month bedaquiline pretomanid linezolid/BPaL regimen without moxifloxacin for RR-TB). Standard of care regimen efficacies (and also costs) were thus a weighted average of efficacies of the first-line and second-line RS or RR regimens with and without component drug resistance (main text Table 2 and Table S1) (7–10).

Table S1: Country-Specific Model Parameters

Parameter	India	South Africa	Philippines	Sources & Notes
Discounted life expectancy at time of TB (years)	24·1 [23·5-25·8]	22·1 [21·4-23·5]	23·2 [21·8-26·3]	Life expectancy by age, weighted by estimated age-TB incidence distribution (11,12) and discounted at 3% annually.
TB case fatality ratio	16% [11-24%]	17% [8-28%]	5% [3-10%]	(12)
Case fatality among those with an unsuccessful treatment outcome	24% [12-36%]	20% [10-30%]	17% [9-26%]	Based on (12), assuming 50% [25-75%] of mortality during treatment is unavoidable through improved regimens.
Percent of RS-TB patients hospitalized	1·0% [0·3-2·1%]	5·0% [2·4-8·5%]	3·0% [2·2-3·9%]	(13,14)
Percent of RR-TB patients hospitalized	25% [19-31%]	30% [24-36%]	6·9% [4·4-9·9%]	
Case detection ratio	73% [53-90%]	59% [43-84%]	67% [43-90%]	(12)
% isoniazid resistance among RS patients	5·3% [4·7-5·9%]	5·8% [4·6-7·1%]	12·9% [10·9-15·2%]	(15–17)
% moxifloxacin resistance among RR patients	16·3% [12·3-20·6%]	13·1% [6·2-22·0%]	1·8% [0·1-6·6%]	(16–18)
Willingness-to-pay thresholds (2021 USD per DALY averted)	$430	$3400	$1060	(19)

Figure S2: Modeled relationship between loss-to-follow-up, duration, and probability of cure

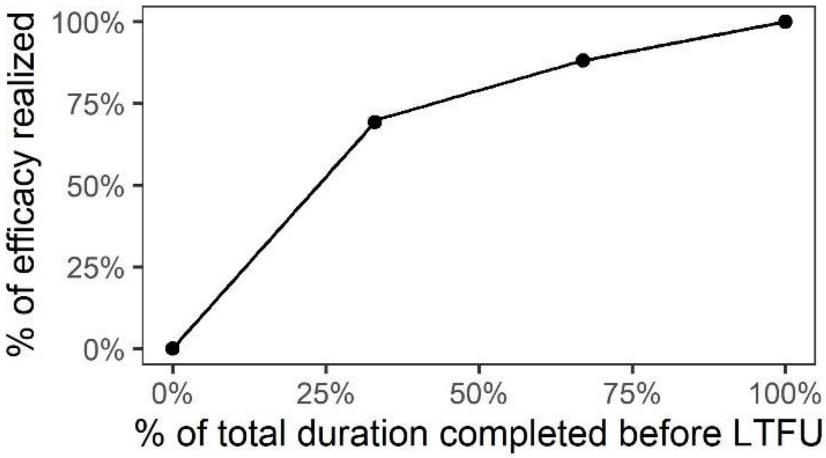

Figure shows the relative risk of cure (% efficacy realized) depending on the percentage of treatment completed before a patient discontinues or is lost-to-follow-up (LTFU). This figure does not include reductions in the probability of cure due to poor adherence while still on treatment, which is modeled separately. This relationship was estimated based on evidence from historical trials of shorter HRZE regimens (20,21) and was fixed across regimens.

Table S2: Disability weights

Health State	Disability Weight	Sources/Notes
Active TB disease	0·33 [0·22-0·45]	(61); same for both rifampin-susceptible and rifampin-resistant TB
Post-TB disability	3·06 [2·02-4·43]	(22); shown cumulative and already discounted
Renal disfunction	0·10 [0·07-0·15]	(61); In the model, each weight was applied for 1 month per person experiencing each adverse event.
Pancreatitis	0·11 [0·08-0·16]	
Anemia	0·05 [0·03-0·08]	
Moderate vision impairment	0·03 [0·02-0·05]	
Arthralgia	0·12 [0·08-0·16]	
Peripheral neuropathy	0·13 [0·09-0·19]	(60,61). In the model, this weight was applied for 3 months for people experiencing short-term peripheral neuropathy and for the lifetime of patients experiencing long-term peripheral neuropathy.
Neutropenia	0	Assumed to be asymptomatic based on (35).
QTcF prolongation	0	
Liver disfunction	0	

All disability weights are shown per year spent with each health state unless otherwise noted. Disability weights did not vary by country.

Figure S3: Estimated serial interval distribution

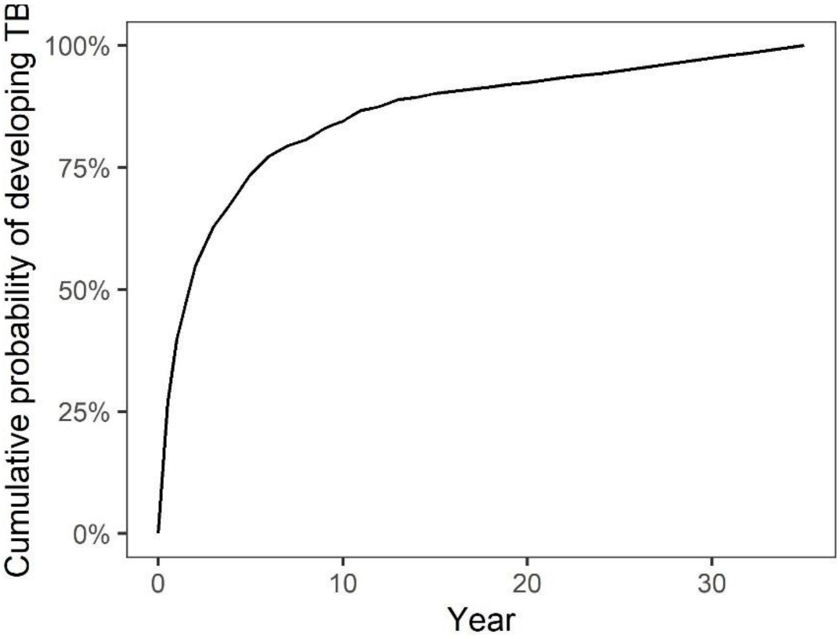

Figure shows the cumulative probability of a secondary case having developed TB disease by year since the index case developed TB disease, conditional on the secondary case eventually developing TB disease. Estimates are based on data reported in (5,22).

Text S2: Additional Cost Analysis Details

The cost analysis included estimation of three price thresholds:

1. **Short-term cost-neutrality**: In this analysis, we estimated the price that would make the novel regimen cost-neutral compared to the standard of care (SOC) considering only costs accrued during a patient's treatment course. Cost-neutrality of a regimen that is more costly based on the cost of drugs alone could be achieved, for example, by reductions in patient care costs through shorter treatment duration or reduced monitoring and side effects.

2. **Medium-term cost-neutrality:** In this analysis, we considered not just cost savings at the individual patient level, but also incorporated estimates of savings from future cases averted, for example by a novel regimen that increases the proportion of patients cured and thus reduces secondary transmission. We estimated the price that would be cost-neutral compared to the SOC on a 5-year time horizon.

3. **Medium-term cost-effectiveness**: This analysis considered the same perspective as the medium-term cost-neutrality analysis (savings during the treatment course and savings from averted future cases and retreatments) but estimated the price at which a novel regimen would be considered cost-effective compared to the SOC under a lifetime horizon. In this analysis, we combined cost estimates with estimates of DALYs associated with active TB, side effects from TB treatment, TB deaths, and post-TB sequelae to estimate a cost-effective drug price using country-specific willingness to pay thresholds (Table S1). The willingness to pay thresholds were sourced from a published study that based thresholds on empirical estimates of health opportunity costs (19). Costs and DALYs were discounted 3% annually.

For RS-TB, the standard of care was assumed to consist of two months of daily isoniazid, rifampin, pyrazinamide, and ethambutol followed by four months of isoniazid and rifampin (6-month HRZE) for most patients. Isoniazid mono-resistant patients receive the 6-month levofloxacin rifampin pyrazinamide ethambutol regimen if their isoniazid-resistance is detected. For RR-TB, the standard of care is assumed to be the 6-month BPaLM regimen (comprising bedaquiline, pretomanid, linezolid, and moxifloxacin); fluoroquinolone-resistant patients receive the 6-month BPaL regimen (no moxifloxacin) if their fluoroquinolone resistance is detected.

In addition to adjusting the efficacy of the SOC regimens to account for isoniazid- and fluoroquinolone-resistance (see details in the appendix describing the modeling of patient cures) we also adjusted the costs. Cost adjustment accounted for the costs of HRZE and Levofloxacin RZE for RS-TB (BPaLM and BPaL for RR-TB), the proportion of patients receiving each regimen, based on the prevalence of and frequency of testing for isoniazid-resistance (fluoroquinolone-resistance for RR-TB) and the costs of isoniazid-resistance testing (fluoroquinolone-resistance testing for RR-TB).

For each SOC and novel regimen, we conducted an ingredients-based costing analysis, which involves multiplying the quantities of different inputs (i.e., services or commodities) needed to deliver the regimen by the country-specific unit costs of each input. Costed inputs included drugs, outpatient treatment and monitoring visits, laboratory tests and diagnostics (including tests related to drug susceptibility, adverse events/side effects/toxicity, and response to treatment), patient support, management of adverse events, and patient-borne travel and time costs. We assumed that, apart from the price of regimen drugs themselves, each country-specific unit cost would be fixed across regimens (Tables S2-S3). However, the quantities of each input required varied by regimen (Table S4). For consistency across countries, the quantities of each input required under the SOC were based on WHO and country-specific TB treatment guidelines and protocols, while the quantities under novel regimens depended on regimen duration and safety. These quantities may therefore better reflect normative treatment costs, since actual treatment of patients in the three countries may deviate from country and global guidelines.

Several quantities scaled with regimen **duration** – including the number of outpatient visits, the amount of treatment support, the number of laboratory tests (i.e., monitoring tests for treatment response and for adverse events/side effects), and the frequency of adverse events. Scaling was often one-to-one but depended on each country's guidelines regarding when visits and lab monitoring should occur (for example, if monitoring occurs only in the first

month of treatment, we assumed that would continue even with shorter regimens). Patient out-of-pocket and indirect costs also varied with duration. The number and type of laboratory tests for adverse events/side effects also scaled with regimen **safety**, as did the monthly incidence of adverse events.

To estimate the savings resulting from fewer treatment failures and relapses, we costed retreatments for those that were not durably cured, occurring an average of 1·65 years after their initial treatment (3). We assumed that 17-24% of uncured patients die during treatment (considering TB-related causes that could be avoided through better treatment regimens only); the remaining uncured patients face country-specific case fatality ratios before being linked to care again (2). Both retreatments and new secondary cases were assumed to spend an average of 8 weeks with TB symptoms before treatment was initiated (23).

To estimate the savings resulting from more secondary cases averted, we estimated the number of future treatments averted by an improved regimen (accounting for transmission averted and for countries' case detection ratios), and for each treatment averted, we costed a full course of treatment. We assumed that each patient who fails treatment or relapses generates an average of 1 secondary case, occurring a median of 1·4 years after being treated, based on (4,5,22) (Figure S2), and that secondary cases are subject to country-specific case fatality ratios (Table S1). The cost-effectiveness analysis considered disability from TB disease, post-TB morbidity and mortality, and adverse events (Table S5), in addition to TB-specific deaths.

All unit costs were converted to 2021 US Dollars (USD) by converting prices reported in USD for earlier years to local currency units (LCU), inflating to 2021 LCU using medical currency consumer price indices (24–26) and converting to 2021 USD using 2021 exchange rates (27).

Table S3: Country-Specific Health System Unit Costs

Cost Component	India		South Africa		Philippines	
	**Estimate**	**Source**	**Estimate**	**Source**	**Estimate**	**Source**
**Outpatient Treatment and Monitoring Visits**						
Outpatient visit	$2·12 [$1·17-3·07]	(28)	$14·83 [$9·85-20·93]	(29)	$3·47 [$2·49-4·58]	(30)
**Laboratory Tests/Screening**						
Pre-initiation drug susceptibility testing (RS)	$29·99 [$20·61-41·16]	(28)	$19·09 [$10·72-30·10]	(31)	$26·91 [$24·77-29·13]	(30)
Sputum smear microscopy	$2·37 [$1·58-3·31]	(28) (32)	$9·02 [$6·78-11·61]	(33) (32)	$5·79 [$3·43-8·72]	(30) (32)
Sputum Culture	$10·26 [$6·50-14·83]		$20·37 [$7·74-41·00]		$27·78 [$22·65-33·47]	
Xpert MTB/RIF and Xpert Ultra	$25·18 [$12·98-41·51]		$25·42 [$19·64-31·71]		$27·74 [$25·22-30·37]	
Xpert XDR	$35·18 [22·98-51·51]		$35·42 [$29·64-41·71]		$37·74 [$35·22-40·37]	
Chest Xray	$3·53 [$2·17-5·25]		$15·79 [$4·13-35·54]		$4·84 [$3·50-6·37]	
Liver function testing	$3·61 [$2·86-4·46]		$9·67 [$5·45-14·95]	(34)	$4·46 [$3·23-5·86]	
Full blood count	$1·16 [$0·73-1·70]		$4·45 [$2·48-6·91]		$3·80 [$2·34-5·66]	
ECG	$1·51 [$0·64-2·76]		$14·61 [$10·70-19·21]	(35)	$5·62 [$2·71-9·50]	
Neuropathy screening	$1·06 [$0·59-1·75]	Clinician time; assumed to be half the cost of an outpatient visit	$7·42 [$4·94-10·41]	Clinician time; assumed to be half the cost of an outpatient visit	$1·74 [$1·24-2·29]	Clinician time; assumed to be half the cost of an outpatient visit
**Adverse Events**						
Liver dysfunction	$154 [$87-240]	(35)	$728 [$408-1139]	(35,36)	$241 [$136-380]	(35)
Pancreatitis	$134 [$78-210]		$472 [$267-734]		$209 [$117-323]	
Anemia	$65 [$36-101]		$97 [$55-151]		$102 [$57-158]	
Neutropenia	$8 [$4-12]		$102 [$58-158]		$13 [$7-19]	
QTcF prolongation	$138 [$77-214]		$517 [$292-810]		$215 [$120-335]	
Renal disfunction	$146 [$82-227]		$619 [$344-961]		$227 [$128-353]	
Vision	$10 [$6-16]	South Africa cost scaled by relative GNI p.c.	$30 [$17-47]		$17 [$10-26]	South Africa cost scaled by relative GNI p.c.
Arthralgia	$5 [$3-7]		$14 [$8-22]		$8 [$4-12]	
Peripheral neuropathy	$0	Only affects DALYs & monitoring costs	$0	Only affects DALYs & monitoring costs	$0	Only affects DALYs & monitoring
**Treatment Support**						
Treatment vouchers (for expenses) per month, RS-TB	$7 [$4-10]	(37)	$0	(38)	$0	Estimate from (14,39)
Treatment vouchers (for expenses) per month, RR-TB	$7 [$4-10]		$134 [$76-210]		$30 [$17-47]	
**Hospitalization (conditional on being hospitalized)**						
Cost per episode, RS-TB	$76 [$51-106]	(28)	$266 [$175-375]	(29)	$134 [$101-172]	(13)
Cost per episode, RR-TB	$400 [$192-689]	(28)	$1819 [$1190-2574]	(29)	$542 [$411-690]	(13)
**Drugs**						
Wastage	8% [5-12%]	(28)	5% [2-8%]	Assumed	5% [2-8%]	(30)

All costs are shown in 2021 USD. This table shows the cost per each cost component (i.e., each service/commodity) listed in the first column. These costs are assumed to be fixed across regimens. The quantities of these cost components required for each regimen are shown in Table S4.

Table S4: Country-Specific Patient-Borne Unit Costs

Cost	India		South Africa		Philippines	
	Estimate	Source	Estimate	Source	Estimate	Source
**Out-of-Pocket Costs**						
Pre-diagnosis (RS)*	$16 [$0-61]	(40)	$22 [$13-35]	(41)	$3 [$1-8]	(13,14)
Pre-diagnosis (RR)*	$32 [$0-122]		$44 [$26-70]		$7 [$0-27]	
Treatment (RS)* per month	$5 [$1-10]*	(40,42–44)	$24 [$2-76]	(41,45,46)	$41 [$14-81]	
Treatment (RR)* per month	$25 [$1-96]	(47)	$53 [$4-167]*	(48)	$155 [$99-225]*	
**Indirect Costs (i.e., productivity/lost wages)**						
Pre-diagnosis (RS)	$83 [23-182]	(40,44)	$48 [$27-75]	(41)	$45 [$26-71]	
Pre-diagnosis (RR)	$166 [46-364]		$96 [$54-150]		$82 [$46-127]	
Treatment intensive phase (RS)	$64 [$17-141] total	(40,42–44,47)	$82 [$46-128] total	(41)	$3 [$2-4] per outpatient visit + $252 [$142-394] per episode of hospitalization	
Treatment continuation phase (RS)	$17 [$5-37] per month		$19 [$11-30] per month			
Treatment all phases (RR)	$103 [$58-161] per month		$61 [$35-96] per month + $219 [$122-340]* per episode of hospital-ization	(48)	$4 [$2-6] per outpatient visit + $1019 [$579-1592]* per episode of hospitalization	

*treatment support voucher costs were subsequently subtracted out of RR-TB out-of-pocket + indirect costs for South Africa and the Philippines and RS-TB costs for India to avoid double-counting. Treatment support vouchers were *not* subtracted out of RR-TB costs for India to avoid underestimation, because the support vouchers are for food and food was not costed in Mullerpattan 2020.

All costs are shown in 2021 USD. This table shows the cost per each cost component (i.e., each service/commodity) listed in the first column. These costs are assumed to be fixed across regimens. The quantities of these cost components required for each regimen are shown in Table S4.

Table S5: Quantities of treatment inputs and events in the cost analysis

Cost Components	Country	SOC RS-TB	TRP-Minimal RS-TB	TRP-Optimal RS-TB	SOC RR-TB	TRP-Minimal RR TB	TRP-Optimal RR-TB	Notes/Sources
**Outpatient Treatment & Monitoring Visits**								
Outpatient visits*	India	8	5	4	9	9	5	-SOC: (49–52). -Novel: scales with duration after month 2
	South Africa	8	5	4	9	9	5	
	Philippines	9	6	5	9	9	5	
**Laboratory Tests**								
Pre-initiation drug susceptibility testing (% patients)	India	20% [13-28%]	20% [13-28%]	20% [13-28%]	36% [27-46%]	36% [27-46%]	36% [27-46%]	-SOC: INH- and FQ-resistance testing from (2) -Novel: same as SOC
	South Africa	50% [22-78%]	50% [22-78%]	50% [22-78%]	50% [22-78%]	50% [22-78%]	50% [22-78%]	
	Philippines	0·3% [0·1-0·7%]	0·3% [0·1-0·7%]	0·3% [0·1-0·7%]	17% [10-25%]	17% [10-25%]	17% [10-25%]	
Sputum smear microscopy*	India	3	3	2	5	5	2	-SOC: (49–53) -Novel: scales with duration after month 2
	South Africa	4	4	2	7	7	3	
	Philippines	4	3	2	7	7	3	
Sputum Culture*	India	0	0	0	3	3	2	
	South Africa	0	0	0	7	7	3	
	Philippines	0	0	0	7	7	3	
Chest Xray*	India	0	0	0	3	3	2	
	South Africa	0	0	0	2	2	2	
	Philippines	0	0	0	2	2	2	
Liver function test (ALT, AST, bilirubin)*	India	0	0	0	7	7	0	-SOC: (49–52,54,55). -Novel: for RR, TRP-optimal monitoring similar to RS SOC & minimal monitoring similar to RR SOC
	South Africa	0	0	0	7	7	0	
	Philippines	0	0	0	7	7	0	
Full blood count*	India	0	0	0	7	7	0	
	South Africa	0	0	0	7	7	0	
	Philippines	0	0	0	7	7	0	
ECG	India	0	0	0	7	7	0	
	South Africa	0	0	0	7	7	0	
	Philippines	0	0	0	7	7	0	
Neuropathy screening	India	0	0	0	7	7	0	
	South Africa	0	0	0	7	7	0	
	Philippines	0	0	0	7	7	0	
**Treatment Support**								
Treatment support vouchers	India	6	4	2	6	6	2	Scales 100% with duration; DS-TB patients in South Africa & Philippines aren't eligible for support vouchers
	South Africa	0	0	0	6	6	2	
	Philippines	0	0	0	6	6	2	
**Adverse Events (% of patients)**								
Liver disfunction	Assumed to be the same across all countries	0·4% [0·1-0·9%]	0·2% [0·1-0·5%]	0·07% [0·02-0·15%]	3·5% [0·9-7·8%]	1·8% [0·4-4·0%]	0·5% [0·1-1·2%]	-SOC DS-TB: (56–59) -SOC DR-TB: (8,10,35,60) -Novel: scales with duration and safety
Pancreatitis		0%	0%	0%	2·0% [0·2-5·5%]	1·0% [0·1-2·8%]	0·3% [0·04-0·9%]	
Anemia		0%	0%	0%	3·0% [0·6-7·1%]	1·5% [0·3-3·5%]	0·5% [0·1-1·1%]	
Neutropenia		0%	0%	0%	4·0% [1·1-8·5%]	2·0% [0·6-4·4%]	0·6% [0·2-1·4%]	
QTcF prolongation		0%	0%	0%	0·5% [0·1-1·3%]	0·25% [0·04-0·64%]	0·08% [0·01-0·20%]	

Renal disfunction		0%	0%	0%	1·0% [0·2-2·4%]	0·5% [0·1-1·2%]	0·15% [0·03-0·37%]	
Vision		0·3% [0·1-0·7%]	0·17% [0·04-0·42%]	0·05% [0·01-0·12%]	0%	0%	0%	
Arthralgia		4·3% [1·2-9·2%]	2·5% [0·7-5·4%]	0·7% [0·2-1·6%]	0%	0%	0%	
Short-term peripheral neuropathy		0%	0%	0%	27% [19-36%]	14·5% [9·9-19·8%]	4·7% [3·1-6·6%]	
Long-term peripheral neuropathy		0%	0%	0%	5% [1·7-9·9%]	2·5% [0·8-5·0%]	0·8% [0·3-1·5%]	

"SOC" = Standard of Care. These quantities were multiplied by the unit costs in tables S2 and S3 to estimate total costs.

Table S6: CHEERS checklist for economic evaluation studies

Topic	No.	Item	Location where item is reported
**Title**	1	Identify the study as an economic evaluation and specify the interventions being compared.	Title
**Abstract**	2	Provide a structured summary that highlights context, key methods, results, and alternative analyses.	Abstract
		**Introduction**	
**Background and objectives**	3	Give the context for the study, the study question, and its practical relevance for decision making in policy or practice.	Introduction, paragraphs 1-5
		**Methods**	
**Health economic analysis plan**	4	Indicate whether a health economic analysis plan was developed and where available.	Not Applicable
**Study population**	5	Describe characteristics of the study population (such as age range, demographics, socioeconomic, or clinical characteristics).	Methods, Model of Patient Outcomes subsection
**Setting and location**	6	Provide relevant contextual information that may influence findings.	Abstract & Methods paragraph 1
**Comparators**	7	Describe the interventions or strategies being compared and why chosen.	Methods paragraphs 1-2
**Perspective**	8	State the perspective(s) adopted by the study and why chosen.	Methods paragraph 1
**Time horizon**	9	State the time horizon for the study and why appropriate.	Methods paragraph 1
**Discount rate**	10	Report the discount rate(s) and reason chosen.	Methods, Price Thresholds subsection
**Selection of outcomes**	11	Describe what outcomes were used as the measure(s) of benefit(s) and harm(s).	Methods, paragraph 1 and Price Thresholds subsection
**Measurement of outcomes**	12	Describe how outcomes used to capture benefit(s) and harm(s) were measured.	Methods, Model of Patient Outcomes and Medium-Term Health Outcomes subsections; Table 2, Text S1; Figures S1-S3; Table S1;
**Valuation of outcomes**	13	Describe the population and methods used to measure and value outcomes.	Methods, Price Thresholds subsection; Tables S1 and S5
**Measurement and valuation of resources and costs**	14	Describe how costs were valued.	Methods, Cost Estimates subsection; Text S2; Tables S2-S4
**Currency, price date, and conversion**	15	Report the dates of the estimated resource quantities and unit costs, plus the currency and year of conversion.	Methods, Cost Estimates subsection
**Rationale and description of model**	16	If modelling is used, describe in detail and why used. Report if the model is publicly available and where it can be accessed.	Methods, Model of Patient Outcomes, Medium-Term Health Outcomes, and Additional Details subsections; Table 2, Text S1; Figures S1-S3; Table S1
**Analytics and assumptions**	17	Describe any methods for analyzing or statistically transforming data, any extrapolation methods, and approaches for validating any model used.	Table 2, Tables S1-S5, Text S1-S2
**Characterizing heterogeneity**	18	Describe any methods used for estimating how the results of the study vary for subgroups.	Not Applicable
**Characterizing distributional effects**	19	Describe how impacts are distributed across different individuals or adjustments made to reflect priority populations.	Not Applicable
**Characterizing uncertainty**	20	Describe methods to characterize any sources of uncertainty in the analysis.	Methods, Price Thresholds subsection
**Approach to engagement with patients and others affected by the study**	21	Describe any approaches to engage patients or service recipients, the general public, communities, or stakeholders (such as clinicians or payers) in the design of the study.	Acknowledgements
		**Results**	
**Study parameters**	22	Report all analytic inputs (such as values, ranges, references) including uncertainty or distributional assumptions.	Table 2, Tables S1-S5, Figures S2-S3
**Summary of main results**	23	Report the mean values for the main categories of costs and outcomes of interest and summarize them in the most appropriate overall measure.	Results text, Figure 1, Table 3, Figure 2

**Effect of uncertainty**	24	Describe how uncertainty about analytic judgments, inputs, or projections affect findings. Report the effect of choice of discount rate and time horizon, if applicable.	Results text, Table 3, Figures S4-S9
**Effect of engagement with patients and others affected by the study**	25	Report on any difference patient/service recipient, general public, community, or stakeholder involvement made to the approach or findings of the study	Not Applicable
**Discussion**			
**Study findings, limitations, generalizability, and current knowledge**	26	Report key findings, limitations, ethical or equity considerations not captured, and how these could affect patients, policy, or practice.	Discussion paragraphs 1, 2, 4, 5, 6
**Other relevant information**			
**Source of funding**	27	Describe how the study was funded and any role of the funder in the identification, design, conduct, and reporting of the analysis	Methods, Role of the Funding Source subsection and Funding Statement
**Conflicts of interest**	28	Report authors conflicts of interest according to journal or International Committee of Medical Journal Editors requirements.	Declaration of Interests and submitted ICMJE forms

From: Husereau et al. 2022. (62)

Supplementary Methods References

1. Imperial MZ, Nahid P, Phillips PPJ, Davies GR, Fielding K, Hanna D, et al. A patient-level pooled analysis of treatment-shortening regimens for drug-susceptible pulmonary tuberculosis. Nat Med. 2018;24(11):1708–15.

2. World Health Organization. Global Tuberculosis Report 2022 [Internet]. 2022 Oct [cited 2022 Dec 21]. Available from: https://www.who.int/publications-detail-redirect/9789240061729

3. Marx FM, Dunbar R, Enarson DA, Williams BG, Warren RM, van der Spuy GD, et al. The Temporal Dynamics of Relapse and Reinfection Tuberculosis After Successful Treatment: A Retrospective Cohort Study. Clin Infect Dis. 2014 Jun 15;58(12):1676–83.

4. Ma Y, Horsburgh CR, White LF, Jenkins HE. Quantifying TB transmission: a systematic review of reproduction number and serial interval estimates for tuberculosis. Epidemiol Infect. 2018 Sep;146(12):1478–94.

5. Borgdorff MW, Sebek M, Geskus RB, Kremer K, Kalisvaart N, van Soolingen D. The incubation period distribution of tuberculosis estimated with a molecular epidemiological approach. Int J Epidemiol. 2011 Aug 1;40(4):964–70.

6. Menzies NA, Swartwood N, Testa C, Malyuta Y, Hill AN, Marks SM, et al. Time Since Infection and Risks of Future Disease for Individuals with Mycobacterium tuberculosis Infection in the United States. Epidemiol Camb Mass. 2021 Jan;32(1):70–8.

7. Gegia M, Winters N, Benedetti A, van Soolingen D, Menzies D. Treatment of isoniazid-resistant tuberculosis with first-line drugs: a systematic review and meta-analysis. Lancet Infect Dis. 2017 Feb 1;17(2):223–34.

8. Conradie F, Bagdasaryan TR, Borisov S, Howell P, Mikiashvili L, Ngubane N, et al. Bedaquiline–Pretomanid–Linezolid Regimens for Drug-Resistant Tuberculosis. N Engl J Med. 2022 Sep 1;387(9):810–23.

9. Conradie F, Diacon AH, Ngubane N, Howell P, Everitt D, Crook AM, et al. Treatment of Highly Drug-Resistant Pulmonary Tuberculosis. N Engl J Med. 2020 Mar 5;382(10):893–902.

10. Nyang'wa BT, Berry C, Kazounis E, Motta I, Parpieva N, Tigay Z, et al. A 24-Week, All-Oral Regimen for Rifampin-Resistant Tuberculosis. N Engl J Med. 2022 Dec 22;387(25):2331–43.

11. World Health Organization. Life tables [Internet]. 2020 [cited 2022 Aug 29]. Available from: https://www.who.int/data/gho/data/themes/topics/indicator-groups/indicator-group-details/GHO/gho-ghe-global-health-estimates-life-tables

12. World Health Organization. Global Tuberculosis Report 2022 [Internet]. 2022 [cited 2023 May 3]. Available from: https://www.who.int/teams/global-tuberculosis-programme/tb-reports/global-tuberculosis-report-2022

13. Florentino JL, Arao RML, Garfin AMC, Gaviola DMG, Tan CR, Yadav RP, et al. Expansion of social protection is necessary towards zero catastrophic costs due to TB: The first national TB patient cost survey in the Philippines. PLOS ONE. 2022 Feb 28;17(2):e0264689.

14. WHO. TB Data [Internet]. 2021 [cited 2022 Aug 15]. Available from: https://www.who.int/teams/global-tuberculosis-programme/data

15. Zignol M, Cabibbe AM, Dean AS, Glaziou P, Alikhanova N, Ama C, et al. Genetic sequencing for surveillance of drug resistance in tuberculosis in highly endemic countries: a multi-country population-based surveillance study. Lancet Infect Dis. 2018 Jun 1;18(6):675–83.

16. National Institute for Communicable Diseases. South African Tuberculosis Drug Resistance Survey [Internet]. [cited 2023 Jun 28]. Available from: https://www.nicd.ac.za/assets/files/K-12750%20NICD%20National%20Survey%20Report_Dev_V11-LR.pdf

17. Indian Ministry of Health and Family Welfare, World Health Organization. Report of the First National Anti-Tuberculosis Drug Resistance Survey [Internet]. 2018 [cited 2023 Feb 14]. Available from: https://tbcindia.gov.in/showfile.php?lid=3315

18. Lim DR, Dean AS, Taguinod-Santiago MR, Borbe-Reyes A, Cabibbe AM, Zignol M, et al. Low prevalence of fluoroquinolone resistance among patients with tuberculosis in the Philippines: results of a national survey. Eur Respir J [Internet]. 2018 Mar 1 [cited 2023 Feb 14];51(3). Available from: https://erj.ersjournals.com/content/51/3/1702571

19. Ochalek J, Lomas J, Claxton K. Estimating health opportunity costs in low-income and middle-income countries: a novel approach and evidence from cross-country data. BMJ Glob Health. 2018 Nov 1;3(6):e000964.

20. Hong Kong Chest Service. A controlled trial of 2-month, 3-month, and 12-month regimens of chemotherapy for sputum-smear-negative pulmonary tuberculosis. Results at 60 months. Am Rev Respir Dis. 1984 Jul;130(1):23–8.

21. Fox W. Whither short-course chemotherapy? Br J Dis Chest. 1981 Oct 1;75(4):331–57.

22. Menzies NA, Quaife M, Allwood BW, Byrne AL, Coussens AK, Harries AD, et al. Lifetime burden of disease due to incident tuberculosis: a global reappraisal including post-tuberculosis sequelae. Lancet Glob Health. 2021 Dec 1;9(12):e1679–87.

23. Teo AKJ, Singh SR, Prem K, Hsu LY, Yi S. Duration and determinants of delayed tuberculosis diagnosis and treatment in high-burden countries: a mixed-methods systematic review and meta-analysis. Respir Res. 2021;22:251.

24. Statistics South Africa. Consumer Price Index data [Internet]. 2022 [cited 2022 Aug 24]. Available from: https://www.statssa.gov.za/?page_id=1854

25. Philippine Statistics Authority. OpenSTAT Database: Consumer Price Index [Internet]. 2022 [cited 2022 Aug 24]. Available from: https://openstat.psa.gov.ph/PXWeb/pxweb/en/DB/DB__2M__PI__CPI__2012/?tablelist=true

26. India Ministry of Statistics and Programme Implementation. Inflation Data [Internet]. 2022 [cited 2022 Aug 24]. Available from: https://www.mospi.gov.in/web/mospi/download-tables-data/-/reports/view/templateOne/16401?q=TBDCAT

27. International Monetary Fund. International Finance Statistics [Internet]. 2022 [cited 2022 Aug 24]. Available from: https://data.imf.org/?sk=4C514D48-B6BA-49ED-8AB9-52B0C1A0179B

28. Chatterjee S, Toshniwal MN, Bhide P, Sachdeva KS, Rao R, Laurence YV, et al. Costs of TB services in India (No 1). Int J Tuberc Lung Dis. 2021 Dec 1;25(12):1013–8.

29. Sinanovic E, Ramma L, Vassall A, Azevedo V, Wilkinson L, Ndjeka N, et al. Impact of reduced hospitalisation on the cost of treatment for drug-resistant tuberculosis in South Africa. Int J Tuberc Lung Dis Off J Int Union Tuberc Lung Dis. 2015 Feb;19(2):172–8.

30. Capeding TPJ, Rosa JD, Lam H, Gaviola DG, Garfin AMC, Hontiveros C, et al. Cost of TB prevention and treatment in the Philippines in 2017. Int J Tuberc Lung Dis. 2022 May;26(5):392–8.

31. Cox H, Ramma L, Wilkinson L, Azevedo V, Sinanovic E. Cost per patient of treatment for rifampicin-resistant tuberculosis in a community-based programme in Khayelitsha, South Africa. Trop Med Int Health. 2015;20(10):1337–45.

32. FIND. GeneXpert® Negotiated Prices [Internet]. 2022 [cited 2022 Aug 31]. Available from: https://www.finddx.org/pricing/genexpert/

33. Vassall A, Siapka M, Foster N, Cunnama L, Ramma L, Fielding K, et al. Cost-effectiveness of Xpert MTB/RIF for tuberculosis diagnosis in South Africa: a real-world cost analysis and economic evaluation. Lancet Glob Health. 2017 Jun 12;5(7):e710–9.

34. South Africa National Health Laboratory Service. State Price List [Internet]. 2018 [cited 2021 Dec 16]. Available from: https://paediatrics.org.za/wp-content/uploads/2023/05/NHLS-State-Price-List-2018.pdf

35. Sweeney S, Berry C, Kazounis E, Motta I, Vassall A, Dodd M, et al. Cost-effectiveness of short, oral treatment regimens for rifampicin resistant tuberculosis. PLOS Glob Public Health. 2022 Dec 7;2(12):e0001337.

36. Schnippel K, Firnhaber C, Berhanu R, Page-Shipp L, Sinanovic E. Direct costs of managing adverse drug reactions during rifampicin-resistant tuberculosis treatment in South Africa. Int J Tuberc Lung Dis. 2018 Apr 1;22(4):393–8.

37. India Ministry of Health and Family Welfare. Nutrition Support to TB Patients (Nikshay Poshan Yojana) [Internet]. 2018 [cited 2022 Aug 30]. Available from: https://tbcindia.gov.in/WriteReadData/l892s/6851513623Nutrition%20support%20DBT%20Scheme%20details.pdf

38. South African Government. Disability grants [Internet]. [cited 2022 Aug 30]. Available from: https://www.gov.za/services/social-benefits/disability-grant

39. The Global Fund. Philippines Global Fund 2020 tuberculosis funding request [Internet]. 2020 [cited 2022 Aug 29]. Available from: https://data.theglobalfund.org/documents

40. Chandra A, Kumar R, Kant S, Krishnan A. Costs of TB care incurred by adult patients with newly diagnosed drug-sensitive TB in Ballabgarh block in northern India. Trans R Soc Trop Med Hyg. 2022 Jan 20;116(1):63–9.

41. Foster N, Vassall A, Cleary S, Cunnama L, Churchyard G, Sinanovic E. The economic burden of TB diagnosis and treatment in South Africa. Soc Sci Med. 2015 Apr 1;130:42–50.

42. Rupani MP, Cattamanchi A, Shete PB, Vollmer WM, Basu S, Dave JD. Costs incurred by patients with drug-susceptible pulmonary tuberculosis in semi-urban and rural settings of Western India. Infect Dis Poverty. 2020 Oct 19;9:144.

43. Sarin R, Vohra V, Singla N, Thomas BE, Krishnan R, Muniyandi M. Identifying costs contributing to catastrophic expenditure among TB patients registered under RNTCP in Delhi metro city in India - ClinicalKey. Indian J Tuberc. 2019 Jan 1;66(1):150–7.

44. Muniyandi M, Thomas BE, Karikalan N, Kannan T, Rajendran K, Saravanan B, et al. Association of Tuberculosis With Household Catastrophic Expenditure in South India. JAMA Netw Open. 2020 Feb 12;3(2):e1920973.

45. Chimbindi N, Bor J, Newell ML, Tanser F, Baltusen R, Hontelez J, et al. Time and money: the true costs of health care utilization for patients receiving ‘free’ HIV/TB care and treatment in rural KwaZulu-Natal. J Acquir Immune Defic Syndr 1999. 2015 Oct 1;70(2):e52–60.

46. Mudzengi D, Sweeney S, Hippner P, Kufa T, Fielding K, Grant AD, et al. The patient costs of care for those with TB and HIV: a cross-sectional study from South Africa. Health Policy Plan. 2017 Nov;32(Suppl 4):iv48–56.

47. Mullerpattan JB, Udwadia ZZ, Banka RA, Ganatra SR, Udwadia ZF. Catastrophic costs of treating drug resistant TB patients in a tertiary care hospital in India. Indian J Tuberc. 2019 Jan 1;66(1):87–91.

48. Ramma L, Cox H, Wilkinson L, Foster N, Cunnama L, Vassall A, et al. Patients' costs associated with seeking and accessing treatment for drug-resistant tuberculosis in South Africa. Int J Tuberc Lung Dis Off J Int Union Tuberc Lung Dis. 2015 Dec 1;19(12):1513–9.

49. India Ministry of Health and Family Welfare. Guidelines for programmatic management of drug resistance tuberculosis in India [Internet]. 2021 [cited 2022 Aug 23]. Available from: https://tbcindia.gov.in/showfile.php?lid=3590

50. Philippines National Tuberculosis Control Program. Manual of Procedures 6th Edition [Internet]. 2022 [cited 2022 Aug 24]. Available from: https://ntp.doh.gov.ph/download/ntp-mop-6th-edition/

51. South Africa National Department of Health. Management of rifampicin-resistance tuberculosis: a clinical reference guide [Internet]. 2019 [cited 2022 Aug 24]. Available from: https://www.health.gov.za/wp-content/uploads/2020/11/management-of-rifampicin-resistant-tb-booklet-0220-v11.pdf

52. South Africa National Department of Health. National tuberculosis management guidelines [Internet]. 2014 [cited 2022 Aug 24]. Available from: https://www.tbonline.info/media/uploads/documents/national_tuberculosis_management_guidelines_%282014%29.pdf

53. World Health Organization India Office. Standards for TB care in India [Internet]. 2014 [cited 2022 Aug 24]. Available from: https://tbcindia.gov.in/showfile.php?lid=3061

54. World Health Organization. Operational Handbook on Tuberculosis, Drug-Susceptible Tuberculosis Treatment [Internet]. 2022 [cited 2022 Aug 29]. Available from: https://www.who.int/publications-detail-redirect/9789240050761

55. World Health Organization. Operational Handbook on Tuberculosis, Drug-Resistant Tuberculosis Treatment [Internet]. 2020 [cited 2022 Aug 29]. Available from: https://www.who.int/publications-detail-redirect/9789240006997

56. Merle CS, Fielding K, Sow OB, Gninafon M, Lo MB, Mthiyane T, et al. A Four-Month Gatifloxacin-Containing Regimen for Treating Tuberculosis. N Engl J Med. 2014 Oct 23;371(17):1588–98.

57. Jindani A, Harrison TS, Nunn AJ, Phillips PPJ, Churchyard GJ, Charalambous S, et al. High-Dose Rifapentine with Moxifloxacin for Pulmonary Tuberculosis. N Engl J Med. 2014 Oct 23;371(17):1599–608.

58. Scott JC, Shah N, Porco T, Flood J. Cost Resulting from Anti-Tuberculosis Drug Shortages in the United States: A Hypothetical Cohort Study. PloS One. 2015;10(8):e0134597.

59. Steele MA, Des Prez RM. The Role of Pyrazinamide in Tuberculosis Chemotherapy. Chest. 1988 Oct 1;94(4):845–50.

60. Conradie F, Diacon AH, Ngubane N, Howell P, Everitt D, Crook AM, et al. Treatment of Highly Drug-Resistant Pulmonary Tuberculosis. N Engl J Med. 2020 Mar 5;382(10):893–902.

61. Global Burden of Disease Collaborative Network. Global Burden of Disease Study 2019 (GBD 2019) Disability Weights [Internet]. Institute for Health Metrics and Evaluation (IHME); 2020 [cited 2021 Aug 10]. Available from: http://ghdx.healthdata.org/record/ihme-data/gbd-2019-disability-weights

62. Husereau D, Drummond M, Augustovski F, Bekker-Grob E de, Briggs AH, Carswell C, et al. Consolidated Health Economic Evaluation Reporting Standards (CHEERS) 2022 Explanation and Elaboration: A Report of the ISPOR CHEERS II Good Practices Task Force. Value Health. 2022 Jan 1;25(1):10–31.

Differences Compared to Modeling in the WHO TRP Document

Text S3: Summary of methodological differences

An earlier iteration of this modeling and cost analysis has been included in the WHO 2023 TRP document. Since that iteration of the analysis, several methodological updates have been made. These updates, and their influence on the results, are summarized below:

1. The price for bedaquiline was reduced to match that published in the August 2023 Global Drug Facility catalog (including 50% free goods). This change resulted in lower costs under the standard of care and lower cost-neutral and cost-effective price thresholds.

2. Uncertainty distributions were added for all model parameters, which due to non-linearities resulted in some differences in mean estimated costs and outcomes (and therefore mean estimated price thresholds).

3. Case fatality and case detection ratios were updated to reflect 2019 estimates from the Global TB Report, reflecting the likelihood that most countries have now returned closer to pre-COVID levels of case detection and quality of treatment outcomes, which would not be reflected in data from 2021 (the latest available as of the time of this analysis). This update improved patient outcomes under the standard of care (including among primary and secondary cases), thus yielding reductions in medium-term cost-neutral and, especially, cost-effective price thresholds.

4. Estimates of patient out-of-pocket and indirect costs were refined based on quality and comparability of evidence across patient cost studies in the published literature. This change affected all cost estimates under the societal perspective (that includes patient-borne costs). The direction of the change varied by country and regimen.

5. Inpatient hospitalization costs at the start of treatment, which were previously excluded because the analysis was focused on incremental costs and hospitalization costs were assumed to be the same across RS- and RR-TB regimens, were added. These costs were still assumed to be the same across RS- and RR-TB regimens, so this change led to an increase in standard of care costs and did not affect price thresholds.

6. Updates were made to parameters governing the prevalence of and testing for isoniazid and fluoroquinolone, based on latest available evidence. These updates had a minor effect on price estimates.

Table S7 compares the present results with those in the TRP document. Taken together these refinements led to some changes to the results but the overall conclusions drawn based on the analyses presented in the WHO document remain unchanged.

Table S7: Comparison of price threshold estimates

Threshold	Perspective	Analysis Iteration	India	Philippines	South Africa
**Regimens for Rifampin-Susceptible TB**					
Short-term cost-neutral	Health system	Published	$70 [$70-80]	$70 [$60-80]	$120 [$100-140]
		Preliminary	$80	$70	$120
	Societal	Published	$140 [$90-210]	$230 [$130-380]	$280 [$180-460]
		Preliminary	$220	$150	$280
Medium-term cost-neutral	Health system	Published	$100 [$80-120]	$100 [$80-120]	$160 [$130-210]
		Preliminary	$110	$110	$170
	Societal	Published	$190 [$130-290]	$320 [$180-530]	$380 [$240-620]
		Preliminary	$320	$240	$420
Cost-effective	Health system	Published	$950 [$510-1740]	$1570 [$800-2900]	$6440 [$3170-12130]
		Preliminary	$1310	$2040	$8680
	Societal	Published	$1050 [$580-1870]	$1800 [$970-3200]	$6660 [$3370-12440]
		Preliminary	$1530	$2180	$8930
**Regimens for Rifampin-Resistant TB**					
Short-term cost-neutral	Health system	Published	$490 [$470-510]	$730 [$670-790]	$1250 [$1030-1520]
		Preliminary	$630	$870	$1400
	Societal	Published	$930 [$720-1230]	$1180 [$980-1430]	$1480 [$1230-1780]
		Preliminary	$830	$880	$1440
Medium-term cost-neutral	Health system	Published	$610 [$550-700]	$960 [$820-1150]	$1680 [$1320-2140]
		Preliminary	$780	$1200	$1820
	Societal	Published	$1200 [$910-1630]	$1610 [$1260-2070]	$2010 [$1590-2560]
		Preliminary	$1080	$1240	$1890
Cost-effective	Health system	Published	$1580 [$1090-2300]	$2700 [$1800-3980]	$8800 [$5350-13820]
		Preliminary	$2020	$3310	$10570
	Societal	Published	$2190 [$1550-3060]	$3370 [$2340-4830]	$9140 [$5640-14230]
		Preliminary	$2320	$3270	$10640

Table shows mean estimated cost-neutral and cost-effective prices per full treatment course of the TRP-Optimal regimens, with 95% uncertainty intervals in brackets. "Published" analysis values match those in Table 3 in the main text, while "Preliminary" analysis values match those in the WHO TRP document. Uncertainty intervals were only estimated in the published analysis.

Supplementary Results

Table S8: Impact of RS-TB regimen improvements on short-term cost-neutral price thresholds

Attribute adjusted	India	South Africa	Philippines
**Drug prices under the standard of care (for reference)**			
None	$46·5 [$46·3-46·7]	$47·2 [$46·5-47·9]	$46·1 [$46·0-46·2]
**Price thresholds of TRP-minimal and TRP-optimal regimens (for reference)**			
TRP-Minimal	$120 [$90-170]	$220 [$150-350]	$190 [$120-300]
TRP-Optimal	$140 [$90-210]	$280 [$180-460]	$230 [$130-380]
**Threshold with single attribute set at TRP-optimal value, with an otherwise standard of care regimen**			
Adherence	$50 [$50-50]	$50 [$50-50]	$50 [$50-50]
Duration	$140 [$90-210]	$280 [$180-460]	$230 [$130-380]
Efficacy	$50 [$50-50]	$50 [$50-50]	$50 [$50-50]
Forgiveness	$50 [$50-50]	$50 [$50-50]	$50 [$50-50]
Safety	$50 [$50-50]	$50 [$50-50]	$50 [$50-50]
**Threshold with single attribute set at standard of care value, with an otherwise TRP-minimal regimen**			
Adherence	$120 [$90-170]	$220 [$150-350]	$190 [$120-300]
Duration	$50 [$50-50]	$50 [$50-50]	$50 [$50-50]
Efficacy	$120 [$90-170]	$220 [$150-350]	$190 [$120-300]
Forgiveness	$120 [$90-170]	$220 [$150-350]	$190 [$120-300]
Safety	$120 [$90-170]	$220 [$150-350]	$190 [$120-300]
**Threshold with single attribute set at TRP-optimal value, with an otherwise TRP-minimal regimen**			
Adherence	$120 [$90-170]	$220 [$150-350]	$190 [$120-300]
Duration	$140 [$90-210]	$280 [$180-460]	$230 [$130-380]
Efficacy	$120 [$90-170]	$220 [$150-350]	$190 [$120-300]
Forgiveness	$120 [$90-170]	$220 [$150-350]	$190 [$120-300]
Safety	$120 [$90-170]	$220 [$150-350]	$190 [$120-300]
**Threshold with single attribute set at standard of care value, with an otherwise TRP-optimal regimen**			
Adherence	$140 [$90-210]	$280 [$180-460]	$230 [$130-380]
Duration	$50 [$50-50]	$50 [$50-50]	$50 [$50-50]
Efficacy	$140 [$90-210]	$280 [$180-460]	$230 [$130-380]
Forgiveness	$140 [$90-210]	$280 [$180-460]	$230 [$130-380]
Safety	$140 [$90-210]	$280 [$180-460]	$230 [$130-380]

Standard of care drug prices represent a weighted average of HRZE and RZE with levofloxacin. Prices therefore differ by slightly country because of differences in drug susceptibility testing patterns and the prevalence of isoniazid resistance among rifampin-susceptible patients. All thresholds are rounded to the nearest ten. Because short-term costs did not include the cost of retreatments and secondary cases, only duration and safety influenced short-term thresholds.

Table S9: Impact of RR-TB regimen improvements on short-term cost-neutral price thresholds

Attribute adjusted	India	South Africa	Philippines
**Drug prices under the standard of care (for reference)**			
None	$431·2 [$430·7-431·6]	$430·3 [$428·4-431·5]	$432·1 [$432·0-432·2]
**Price thresholds of TRP-minimal and TRP-optimal regimens (for reference)**			
TRP-Minimal	$440 [$430-440]	$460 [$440-480]	$440 [$440-450]
TRP-Optimal	$930 [$720-1230]	$1480 [$1230-1780]	$1180 [$980-1430]
**Threshold with single attribute set at TRP-optimal value, with an otherwise standard of care regimen**			
Adherence	$430 [$430-430]	$430 [$430-430]	$430 [$430-430]
Duration	$910 [$710-1210]	$1380 [$1140-1690]	$1140 [$940-1390]
Efficacy	$430 [$430-430]	$430 [$430-430]	$430 [$430-430]
Forgiveness	$430 [$430-430]	$430 [$430-430]	$430 [$430-430]
Safety	$480 [$480-500]	$700 [$650-750]	$550 [$520-580]
**Threshold with single attribute set at standard of care value, with an otherwise TRP-minimal regimen**			
Adherence	$440 [$430-440]	$460 [$440-480]	$440 [$440-450]
Duration	$440 [$430-440]	$460 [$440-480]	$440 [$440-450]
Efficacy	$440 [$430-440]	$460 [$440-480]	$440 [$440-450]
Forgiveness	$440 [$430-440]	$460 [$440-480]	$440 [$440-450]
Safety	$430 [$430-430]	$430 [$430-430]	$430 [$430-430]
**Threshold with single attribute set at TRP-optimal value, with an otherwise TRP-minimal regimen**			
Adherence	$440 [$430-440]	$460 [$440-480]	$440 [$440-450]
Duration	$910 [$710-1210]	$1380 [$1140-1690]	$1140 [$940-1390]
Efficacy	$440 [$430-440]	$460 [$440-480]	$440 [$440-450]
Forgiveness	$440 [$430-440]	$460 [$440-480]	$440 [$440-450]
Safety	$480 [$480-500]	$700 [$650-750]	$550 [$520-580]
**Threshold with single attribute set at standard of care value, with an otherwise TRP-optimal regimen**			
Adherence	$930 [$720-1230]	$1480 [$1230-1780]	$1180 [$980-1430]
Duration	$480 [$480-500]	$700 [$650-750]	$550 [$520-580]
Efficacy	$930 [$720-1230]	$1480 [$1230-1780]	$1180 [$980-1430]
Forgiveness	$930 [$720-1230]	$1480 [$1230-1780]	$1180 [$980-1430]
Safety	$910 [$700-1210]	$1380 [$1130-1680]	$1140 [$930-1380]

Standard of care drug prices represent a weighted average of BPaL with and without moxifloxacin. Prices therefore differ by slightly country because of differences in drug susceptibility testing patterns and the prevalence of fluoroquinolone resistance. All results are rounded to the nearest 10. Because short-term costs did not include the cost of retreatments and secondary cases, only duration and safety influenced short-term thresholds.

Table S10: Impact of RS-TB regimen improvements on medium-term cost-neutral price thresholds

Attribute adjusted	India	South Africa	Philippines
**Drug prices under the standard of care (for reference)**			
None	$46·5 [$46·3-46·7]	$47·2 [$46·5-47·9]	$46·1 [$46·0-46·2]
**Price thresholds of TRP-minimal and TRP-optimal regimens (for reference)**			
TRP-Minimal	$140 [$100-190]	$250 [$170-400]	$210 [$130-340]
TRP-Optimal	$190 [$130-290]	$380 [$240-620]	$320 [$180-530]
**Threshold with single attribute set at TRP-optimal value, with an otherwise standard of care regimen**			
Adherence	$90 [$60-140]	$120 [$70-210]	$110 [$70-200]
Duration	$140 [$90-210]	$280 [$190-470]	$240 [$130-390]
Efficacy	$60 [$50-60]	$70 [$60-80]	$60 [$50-70]
Forgiveness	$60 [$50-90]	$80 [$60-120]	$70 [$50-110]
Safety	$50 [$50-50]	$50 [$50-50]	$50 [$50-50]
**Threshold with single attribute set at standard of care value, with an otherwise TRP-minimal regimen**			
Adherence	$140 [$100-190]	$250 [$170-400]	$210 [$130-340]
Duration	$60 [$50-70]	$70 [$50-100]	$70 [$50-90]
Efficacy	$140 [$100-190]	$250 [$170-400]	$210 [$130-340]
Forgiveness	$120 [$90-170]	$220 [$150-360]	$190 [$120-300]
Safety	$140 [$100-190]	$250 [$170-400]	$210 [$130-340]
**Threshold with single attribute set at TRP-optimal value, with an otherwise TRP-minimal regimen**			
Adherence	$170 [$110-250]	$300 [$190-500]	$270 [$160-440]
Duration	$150 [$100-230]	$300 [$200-500]	$250 [$140-420]
Efficacy	$150 [$110-210]	$270 [$180-430]	$230 [$140-370]
Forgiveness	$140 [$100-200]	$260 [$170-420]	$220 [$140-360]
Safety	$140 [$100-190]	$250 [$170-400]	$210 [$130-340]
**Threshold with single attribute set at standard of care value, with an otherwise TRP-optimal regimen**			
Adherence	$170 [$110-250]	$330 [$220-550]	$280 [$160-460]
Duration	$100 [$70-160]	$140 [$90-250]	$130 [$80-240]
Efficacy	$180 [$120-270]	$350 [$230-590]	$300 [$170-490]
Forgiveness	$190 [$130-290]	$380 [$240-620]	$320 [$180-530]
Safety	$190 [$130-290]	$380 [$240-620]	$320 [$180-530]

Standard of care drug prices represent a weighted average of HRZE and RZE with levofloxacin. Prices therefore differ by slightly country because of differences in drug susceptibility testing patterns and the prevalence of isoniazid resistance among rifampin-susceptible patients. All thresholds are rounded to the near ten.

Table S11: Impact of RR-TB regimen improvements on medium-term cost-neutral price thresholds

Attribute adjusted	India	South Africa	Philippines
**Drug prices under the standard of care (for reference)**			
None	$431·2 [$430·7-431·6]	$430·3 [$428·4-431·5]	$432·1 [$432·0-432·2]
**Price thresholds of TRP-minimal and TRP-optimal regimens (for reference)**			
TRP-Minimal	$470 [$450-510]	$530 [$480-610]	$500 [$460-570]
TRP-Optimal	$1200 [$910-1630]	$2010 [$1590-2560]	$1610 [$1260-2070]
**Threshold with single attribute set at TRP-optimal value, with an otherwise standard of care regimen**			
Adherence	$580 [$480-770]	$720 [$530-1090]	$670 [$510-950]
Duration	$920 [$710-1220]	$1420 [$1170-1740]	$1180 [$970-1440]
Efficacy	$530 [$460-630]	$620 [$490-810]	$590 [$480-730]
Forgiveness	$480 [$450-550]	$540 [$470-660]	$520 [$460-620]
Safety	$480 [$470-500]	$700 [$650-760]	$550 [$520-590]
**Threshold with single attribute set at standard of care value, with an otherwise TRP-minimal regimen**			
Adherence	$430 [$430-450]	$460 [$440-480]	$450 [$430-470]
Duration	$470 [$450-510]	$530 [$480-610]	$500 [$460-570]
Efficacy	$470 [$450-510]	$530 [$480-610]	$500 [$460-570]
Forgiveness	$470 [$450-510]	$530 [$480-610]	$500 [$460-570]
Safety	$460 [$440-510]	$500 [$460-590]	$490 [$450-560]
**Threshold with single attribute set at TRP-optimal value, with an otherwise TRP-minimal regimen**			
Adherence	$590 [$480-780]	$750 [$550-1110]	$680 [$520-960]
Duration	$950 [$740-1260]	$1490 [$1220-1820]	$1230 [$1000-1510]
Efficacy	$570 [$500-680]	$730 [$570-930]	$660 [$540-810]
Forgiveness	$490 [$450-560]	$570 [$490-710]	$540 [$470-640]
Safety	$520 [$490-560]	$770 [$690-870]	$610 [$560-680]
**Threshold with single attribute set at standard of care value, with an otherwise TRP-optimal regimen**			
Adherence	$1090 [$840-1450]	$1800 [$1460-2210]	$1450 [$1170-1780]
Duration	$760 [$610-1010]	$1230 [$940-1680]	$980 [$750-1340]
Efficacy	$1080 [$820-1460]	$1780 [$1430-2260]	$1430 [$1130-1830]
Forgiveness	$1200 [$910-1630]	$2010 [$1590-2560]	$1610 [$1260-2070]
Safety	$1180 [$890-1610]	$1910 [$1490-2460]	$1570 [$1220-2030]

Standard of care drug prices represent a weighted average of BPaL with and without moxifloxacin. Prices therefore differ by slightly country because of differences in drug susceptibility testing patterns and the prevalence of fluoroquinolone resistance. All results are rounded to the nearest 10.

Table S12: Impact of RS-TB regimen improvements on cost-effective price thresholds

Attribute adjusted	India	South Africa	Philippines
**Drug prices under the standard of care (for reference)**			
None	$46·5 [$46·3-46·7]	$47·2 [$46·5-47·9]	$46·1 [$46·0-46·2]
**Price thresholds of TRP-minimal and TRP-optimal regimens (for reference)**			
TRP-Minimal	$400 [$250-660]	$2170 [$1170-3980]	$660 [$410-1130]
TRP-Optimal	$1050 [$580-1870]	$6660 [$3370-12440]	$1800 [$970-3200]
**Threshold with single attribute set at TRP-optimal value, with an otherwise standard of care regimen**			
Adherence	$660 [$250-1400]	$4270 [$1430-9460]	$1080 [$380-2300]
Duration	$230 [$170-310]	$970 [$740-1270]	$420 [$300-590]
Efficacy	$200 [$140-280]	$1120 [$680-1690]	$300 [$200-440]
Forgiveness	$310 [$130-630]	$1860 [$630-4090]	$460 [$190-1010]
Safety	$50 [$50-50]	$50 [$50-50]	$50 [$50-50]
**Threshold with single attribute set at standard of care value, with an otherwise TRP-minimal regimen**			
Adherence	$400 [$250-660]	$2170 [$1170-3980]	$660 [$410-1130]
Duration	$230 [$100-450]	$1290 [$430-2850]	$330 [$140-720]
Efficacy	$400 [$250-660]	$2170 [$1170-3980]	$660 [$410-1130]
Forgiveness	$190 [$150-240]	$730 [$550-940]	$330 [$240-450]
Safety	$400 [$250-660]	$2170 [$1170-3980]	$660 [$410-1130]
**Threshold with single attribute set at TRP-optimal value, with an otherwise TRP-minimal regimen**			
Adherence	$900 [$420-1760]	$5650 [$2340-11690]	$1530 [$710-2960]
Duration	$410 [$270-640]	$2220 [$1320-3770]	$700 [$460-1120]
Efficacy	$590 [$400-890]	$3540 [$2140-5620]	$900 [$650-1520]
Forgiveness	$490 [$280-870]	$2840 [$1400-5440]	$790 [$470-1460]
Safety	$400 [$250-660]	$2180 [$1170-3980]	$660 [$410-1130]
**Threshold with single attribute set at standard of care value, with an otherwise TRP-optimal regimen**			
Adherence	$670 [$440-1030]	$4020 [$2430-6530]	$1190 [$740-1770]
Duration	$870 [$400-1690]	$5700 [$2430-11490]	$1420 [$620-2800]
Efficacy	$840 [$430-1580]	$5210 [$2370-10420]	$1450 [$730-2690]
Forgiveness	$1050 [$580-1870]	$6660 [$3370-12440]	$1800 [$970-3200]
Safety	$1050 [$580-1870]	$6660 [$3370-12440]	$1800 [$970-3200]

Standard of care drug prices represent a weighted average of HRZE and RZE with levofloxacin. Prices therefore differ slightly by country because of differences in drug susceptibility testing patterns and the prevalence of isoniazid resistance among rifampin-susceptible patients.

Table S13: Impact of RR-TB regimen improvements on cost-effective price thresholds

Attribute adjusted	India	South Africa	Philippines
**Drug prices under the standard of care (for reference)**			
None	\$431·2 [\$430·7-431·6]	\$430·3 [\$428·4-431·5]	\$432·1 [\$432·0-432·2]
**Price thresholds of TRP-minimal and TRP-optimal regimens (for reference)**			
TRP-Minimal	\$630 [\$520-800]	\$1640 [\$990-2710]	\$790 [\$600-1090]
TRP-Optimal	\$2190 [\$1550-3060]	\$9140 [\$5640-14230]	\$3370 [\$2340-4830]
**Threshold with single attribute set at TRP-optimal value, with an otherwise standard of care regimen**			
Adherence	\$1080 [\$650-1850]	\$4280 [\$1700-9030]	\$1560 [\$790-2810]
Duration	\$1070 [\$860-1380]	\$2540 [\$2080-3110]	\$1510 [\$1260-1820]
Efficacy	\$850 [\$560-1240]	\$2910 [\$1170-5370]	\$1120 [\$640-1790]
Forgiveness	\$660 [\$510-930]	\$1800 [\$890-3470]	\$810 [\$560-1270]
Safety	\$520 [\$490-560]	\$970 [\$790-1270]	\$650 [\$580-750]
**Threshold with single attribute set at standard of care value, with an otherwise TRP-minimal regimen**			
Adherence	\$470 [\$450-510]	\$730 [\$550-1020]	\$540 [\$480-640]
Duration	\$630 [\$520-800]	\$1640 [\$990-2710]	\$790 [\$600-1090]
Efficacy	\$630 [\$520-800]	\$1640 [\$990-2710]	\$790 [\$600-1090]
Forgiveness	\$630 [\$520-800]	\$1640 [\$990-2710]	\$790 [\$600-1090]
Safety	\$590 [\$490-750]	\$1360 [\$760-2390]	\$690 [\$530-980]
**Threshold with single attribute set at TRP-optimal value, with an otherwise TRP-minimal regimen**			
Adherence	\$1120 [\$690-1890]	\$4570 [\$1960-9300]	\$1650 [\$870-2920]
Duration	\$1210 [\$960-1550]	\$3380 [\$2630-4460]	\$1740 [\$1420-2150]
Efficacy	\$1070 [\$750-1470]	\$4270 [\$2300-6780]	\$1500 [\$970-2200]
Forgiveness	\$720 [\$550-1010]	\$2210 [\$1190-4020]	\$940 [\$660-1440]
Safety	\$670 [\$570-840]	\$1890 [\$1230-2960]	\$890 [\$710-1200]
**Threshold with single attribute set at standard of care value, with an otherwise TRP-optimal regimen**			
Adherence	\$1720 [\$1310-2240]	\$6400 [\$4330-9010]	\$2390 [\$1950-3370]
Duration	\$1700 [\$1080-2580]	\$7970 [\$4190-13480]	\$2610 [\$1540-4110]
Efficacy	\$1690 [\$1200-2460]	\$6190 [\$3730-10570]	\$2550 [\$1790-3800]
Forgiveness	\$2190 [\$1550-3060]	\$9140 [\$5640-14230]	\$3370 [\$2340-4830]
Safety	\$2160 [\$1520-3030]	\$8970 [\$5460-14030]	\$3300 [\$2280-4750]

Standard of care drug prices represent a weighted average of BPaL with and without moxifloxacin. Prices therefore differ slightly by country because of differences in drug susceptibility testing patterns and the prevalence of fluoroquinolone resistance among rifampin-resistant patients.

Table S14: Impact of RS-TB regimen improvements on DALYs and percent of patients durably cured

Attribute adjusted	Cures – pooled*	DALYs - India	DALYs - South Africa	DALYs - Philippines
**Outcomes under the standard of care regimen**				
None	83% [70-91%]	5·7 [3·9-8·1]	5·5 [3·7-7·7]	4·9 [3·3-6·9]
**Outcomes under TRP-minimal and TRP-optimal regimens**				
TRP-Minimal	87% [77-93%]	5·0 [3·5-6·9]	4·8 [3·3-6·7]	4·4 [3·0-6·1]
TRP-Optimal	98% [98-99%]	3·3 [2·2-4·7]	3·3 [2·2-4·6]	3·3 [2·2-4·6]
**Outcomes with single attribute set at TRP-optimal value, with an otherwise standard of care regimen**				
Adherence	94% [92-95%]	4·1 [2·9-5·4]	4·0 [2·8-5·4]	3·8 [2·6-5·2]
Duration	84% [71-92%]	5·4 [3·7-7·8]	5·2 [3·5-7·4]	4·7 [3·1-6·6]
Efficacy	86% [73-93%]	5·2 [3·5-7·6]	5·1 [3·4-7·2]	4·6 [3·1-6·5]
Forgiveness	88% [80-93%]	4·9 [3·5-6·7]	4·8 [3·4-6·5]	4·4 [3·0-6·0]
Safety	83% [70-91%]	5·7 [3·9-8·1]	5·5 [3·7-7·7]	4·9 [3·3-6·9]
**Outcomes with single attribute set at standard of care value, with an otherwise TRP-minimal regimen**				
Adherence	87% [77-93%]	5·0 [3·5-6·9]	4·8 [3·3-6·7]	4·4 [3-6·1·0]
Duration	86% [77-92%]	5·2 [3·7-7·1]	5·0 [3·5-6·8]	4·5 [3·1-6·2]
Efficacy	87% [77-93%]	5·0 [3·5-6·9]	4·8 [3·3-6·7]	4·4 [3·0-6·1]
Forgiveness	83% [70-91%]	5·5 [3·8-7·9]	5·3 [3·6-7·5]	4·7 [3·2-6·7]
Safety	87% [77-93%]	5·0 [3·5-6·9]	4·8 [3·3-6·7]	4·4 [3·0-6·1]
**Outcomes with single attribute set at TRP-optimal value, with an otherwise TRP-minimal regimen**				
Adherence	94% [93-96%]	3·9 [2·8-5·3]	3·8 [2·7-5·2]	3·7 [2·5-5·0]
Duration	87% [78-93%]	4·9 [3·4-6·8]	4·7 [3·2-6·6]	4·3 [2·9-6·0]
Efficacy	90% [81-95%]	4·5 [3·1-6·4]	4·4 [3·0-6·2]	4·1 [2·8-5·7]
Forgiveness	88% [80-93%]	4·8 [3·4-6·5]	4·6 [3·2-6·3]	4·2 [2·9-5·8]
Safety	87% [77-93%]	5·0 [3·5-6·9]	4·8 [3·3-6·7]	4·4 [3·0-6·1]
**Outcomes with single attribute set at standard of care value, with an otherwise TRP-optimal regimen**				
Adherence	92% [85-96%]	4·2 [2·9-5·8]	4·1 [2·8-5·7]	3·8 [2·6-5·3]
Duration	97% [96-98%]	3·6 [2·5-4·9]	3·6 [2·4-4·9]	3·5 [2·4-4·8]
Efficacy	95% [93-96%]	3·8 [2·7-5·2]	3·7 [2·6-5·1]	3·6 [2·4-4·9]
Forgiveness	98% [98-99%]	3·3 [2·2-4·7]	3·3 [2·2-4·6]	3·3 [2·2-4·6]
Safety	98% [98-99%]	3·3 [2·2-4·7]	3·3 [2·2-4·6]	3·3 [2·2-4·6]

Cures were very similar in all 3 countries and are therefore shown pooled across countries. DALYs varied more across countries due to different case fatality ratios and case detection ratios, which affected DALYs attributed to retreatments and secondary cases.

Table S15: Impact of RR-TB regimen improvements on DALYs and percent of patients durably cured

Attribute adjusted	Cures – pooled*	DALYs - India	DALYs - South Africa	DALYs - Philippines
**Outcomes under the standard of care regimen**				
None	78% [67-87%]	6·6 [4·7-9·0]	6·3 [4·4-8·5]	5·6 [3·9-7·6]
**Outcomes under TRP-minimal and TRP-optimal regimens**				
TRP-Minimal	81% [71-88%]	6·1 [4·4-8·2]	5·8 [4·1-7·8]	5·2 [3·7-7·1]
TRP-Optimal	96% [96-97%]	3·6 [2·5-5·0]	3·6 [2·5-4·9]	3·5 [2·3-4·8]
**Outcomes with single attribute set at TRP-optimal value, with an otherwise standard of care regimen**				
Adherence	88% [82-93%]	5·1 [3·7-6·8]	4·9 [3·5-6·6]	4·6 [3·2-6·2]
Duration	79% [68-88%]	6·1 [4·2-8·4]	5·8 [4·0-8·0]	5·1 [3·5-7·1]
Efficacy	85% [73-92%]	5·6 [3·9-7·8]	5·4 [3·8-7·5]	4·9 [3·4-6·8]
Forgiveness	82% [73-89%]	6·0 [4·4-8·0]	5·8 [4·1-7·7]	5·2 [3·7-7·0]
Safety	78% [67-87%]	6·5 [4·6-8·8]	6·2 [4·3-8·4]	5·5 [3·8-7·5]
**Outcomes with single attribute set at standard of care value, with an otherwise TRP-minimal regimen**				
Adherence	78% [67-87%]	6·5 [4·6-8·8]	6·2 [4·3-8·4]	5·5 [3·8-7·5]
Duration	81% [71-88%]	6·1 [4·4-8·2]	5·8 [4·1-7·8]	5·2 [3·7-7·1]
Efficacy	81% [71-88%]	6·1 [4·4-8·2]	5·8 [4·1-7·8]	5·2 [3·7-7·1]
Forgiveness	81% [71-88%]	6·1 [4·4-8·2]	5·8 [4·1-7·8]	5·2 [3·7-7·1]
Safety	81% [71-88%]	6·2 [4·5-8·3]	5·9 [4·2-7·9]	5·3 [3·8-7·2]
**Outcomes with single attribute set at TRP-optimal value, with an otherwise TRP-minimal regimen**				
Adherence	88% [82-93%]	5·0 [3·6-6·7]	4·8 [3·4-6·5]	4·5 [3·1-6·1]
Duration	82% [72-89%]	5·7 [4-7·7·0]	5·5 [3·8-7·4]	4·9 [3·4-6·7]
Efficacy	87% [78-93%]	5·1 [3·6-7·0]	4·9 [3·5-6·7]	4·5 [3·2-6·2]
Forgiveness	82% [74-89%]	5·9 [4·3-7·8]	5·6 [4·0-7·5]	5·0 [3·6-6·8]
Safety	81% [71-88%]	6·1 [4·4-8·2]	5·8 [4·1-7·8]	5·2 [3·7-7·1]
**Outcomes with single attribute set at standard of care value, with an otherwise TRP-optimal regimen**				
Adherence	90% [82-94%]	4·5 [3·2-6·2]	4·4 [3·1-6·0]	4·1 [2·8-5·6]
Duration	95% [94-96%]	4·0 [2·8-5·3]	3·9 [2·8-5·3]	3·8 [2·6-5·1]
Efficacy	89% [83-94%]	4·6 [3·2-6·3]	4·5 [3·1-6·1]	4·1 [2·8-5·7]
Forgiveness	96% [96-97%]	3·6 [2·5-5·0]	3·6 [2·5-4·9]	3·5 [2·3-4·8]
Safety	96% [96-97%]	3·7 [2·5-5·0]	3·6 [2·5-5·0]	3·5 [2·4-4·8]

Cures were very similar in all 3 countries and are therefore shown pooled across countries. DALYs varied more across countries due to different case fatality ratios and case detection ratios, which affected DALYs attributed to retreatments and secondary cases.

Figure S4: Cost-effective prices with variation in willingness-to-pay: India, societal perspective

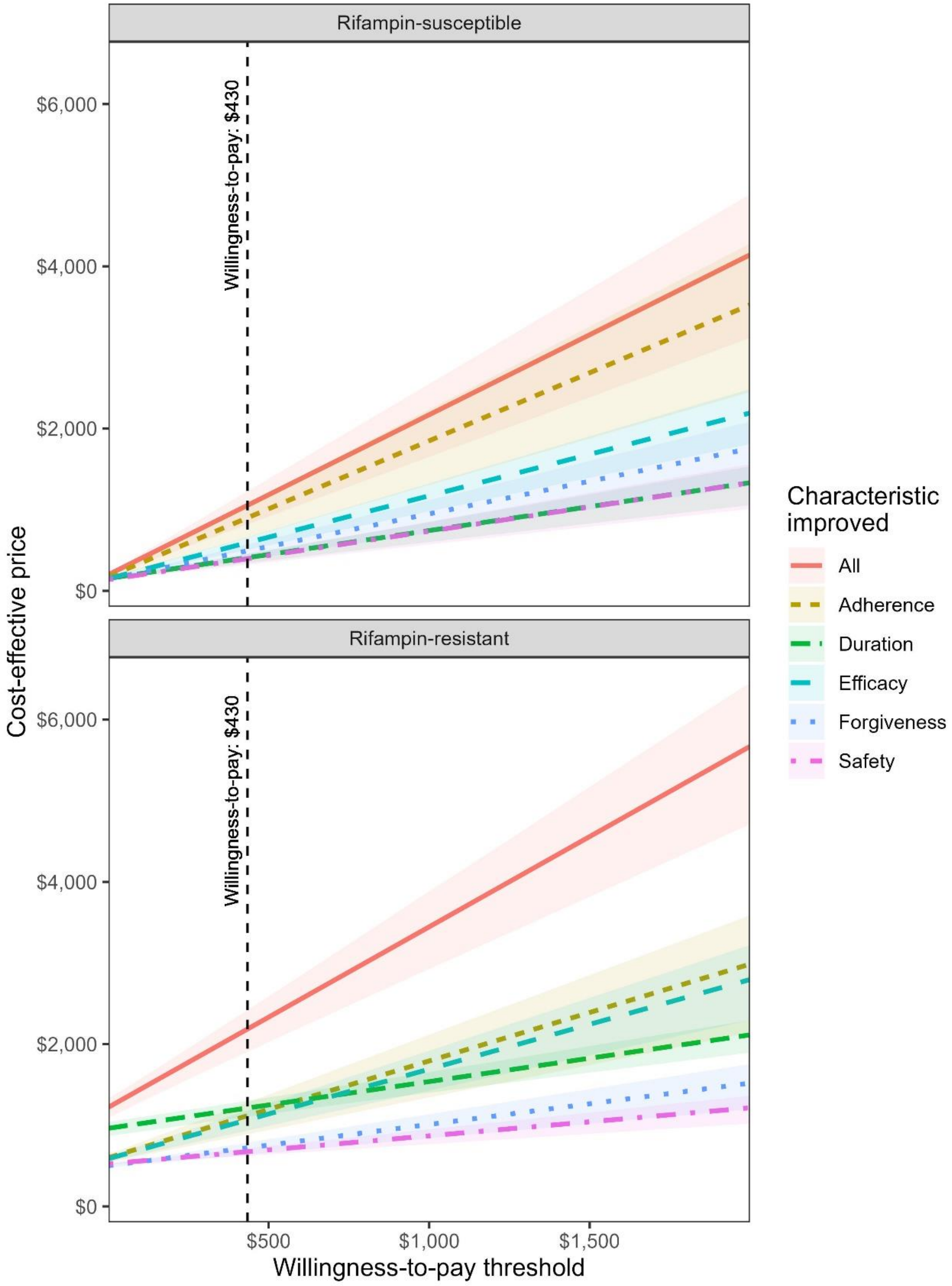

Figure shows how the cost-effective price thresholds (y-axis) for TRP-minimal rifampin-susceptible (RS) and rifampin-resistant (RR) regimens in India vary with the willingness-to-pay threshold (x-axis) when 1 characteristic at a time is set to its "optimal" value in the TRPs (yellow through purple), and when all characteristics are set to their "optimal" values (red="TRP-Optimal" regimen). The willingness-to-pay threshold used in the main analysis is shown with a dashed vertical line. Shaded areas indicate 95% uncertainty intervals around the means (lines).

Figure S5: Cost-effective prices with variation in willingness-to-pay: India, health systems perspective

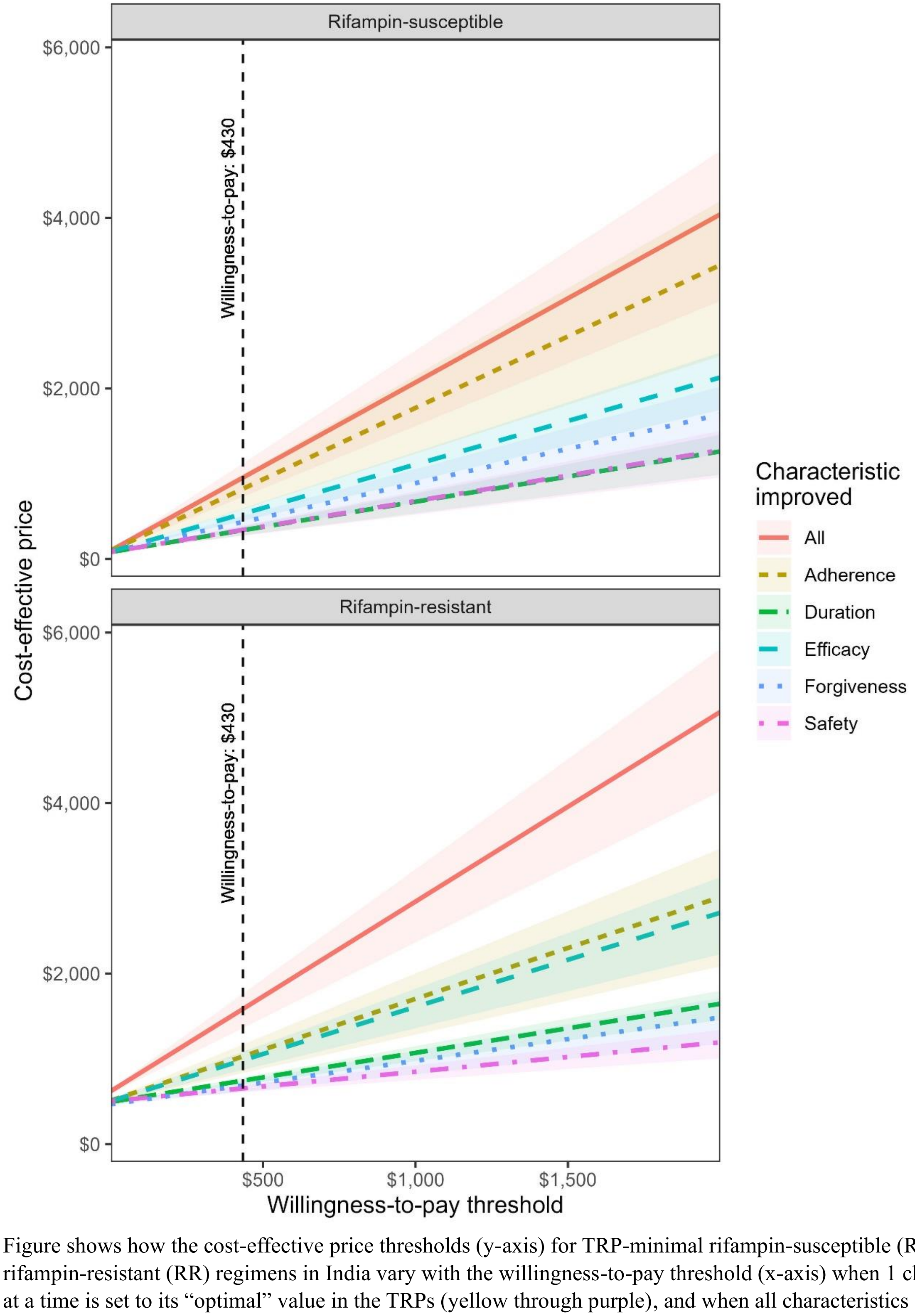

Figure shows how the cost-effective price thresholds (y-axis) for TRP-minimal rifampin-susceptible (RS) and rifampin-resistant (RR) regimens in India vary with the willingness-to-pay threshold (x-axis) when 1 characteristic at a time is set to its "optimal" value in the TRPs (yellow through purple), and when all characteristics are set to their "optimal" values (red="TRP-Optimal" regimen). The willingness-to-pay threshold used in the main analysis is shown with a dashed vertical line. Shaded areas indicate 95% uncertainty intervals around the means (lines).

Figure S6: Cost-effective prices with variation in willingness-to-pay: South Africa, societal perspective

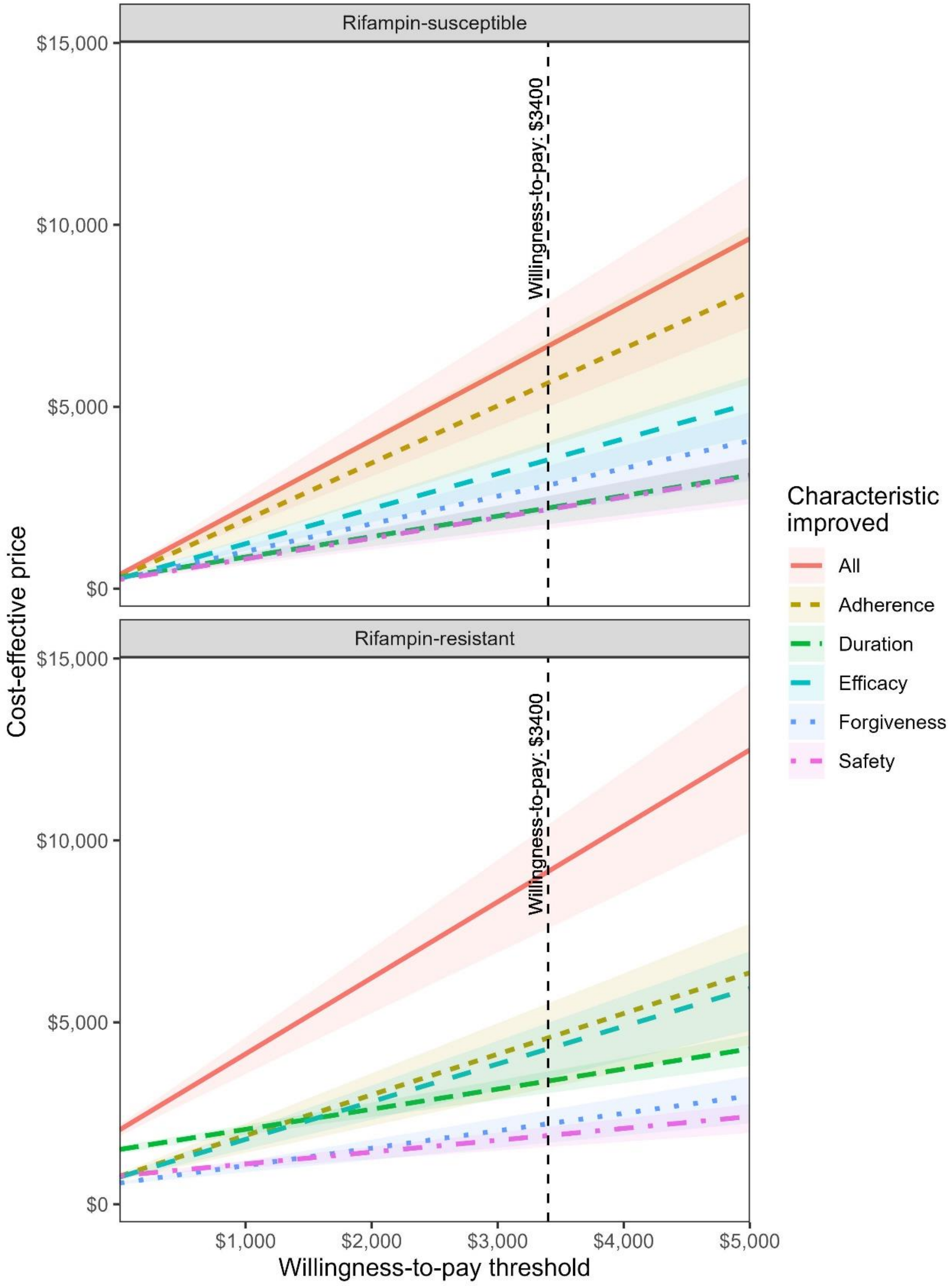

Figure shows how the cost-effective price thresholds (y-axis) for TRP-minimal rifampin-susceptible (RS) and rifampin-resistant (RR) regimens in South Africa vary with the willingness-to-pay threshold (x-axis) when 1 characteristic at a time is set to its “optimal” value in the TRPs (yellow through purple), and when all characteristics are set to their “optimal” values (red=”TRP-Optimal” regimen). The willingness-to-pay threshold used in the main analysis is shown with a dashed vertical line. Shaded areas indicate 95% uncertainty intervals around the means.

Figure S7: Cost-effective prices with variation in willingness-to-pay: South Africa, health systems perspective

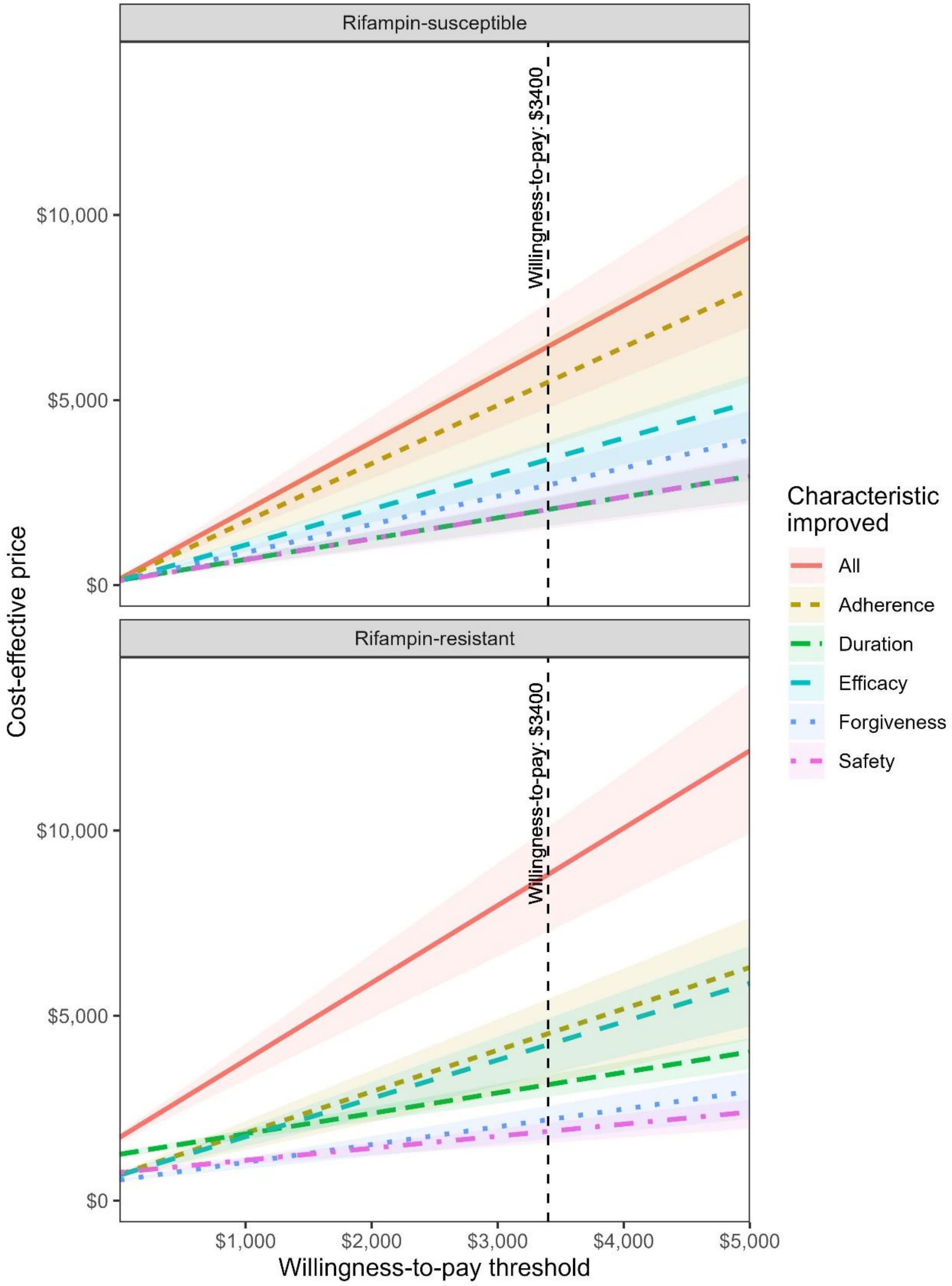

Figure shows how the cost-effective price thresholds (y-axis) for TRP-minimal rifampin-susceptible (RS) and rifampin-resistant (RR) regimens in South Africa vary with the willingness-to-pay threshold (x-axis) when 1 characteristic at a time is set to its "optimal" value in the TRPs (yellow through purple), and when all characteristics are set to their "optimal" values (red="TRP-Optimal" regimen). The willingness-to-pay threshold used in the main analysis is shown with a dashed vertical line. Shaded areas indicate 95% uncertainty intervals around the means.

Figure S8: Cost-effective prices with variation in willingness-to-pay: Philippines, societal perspective

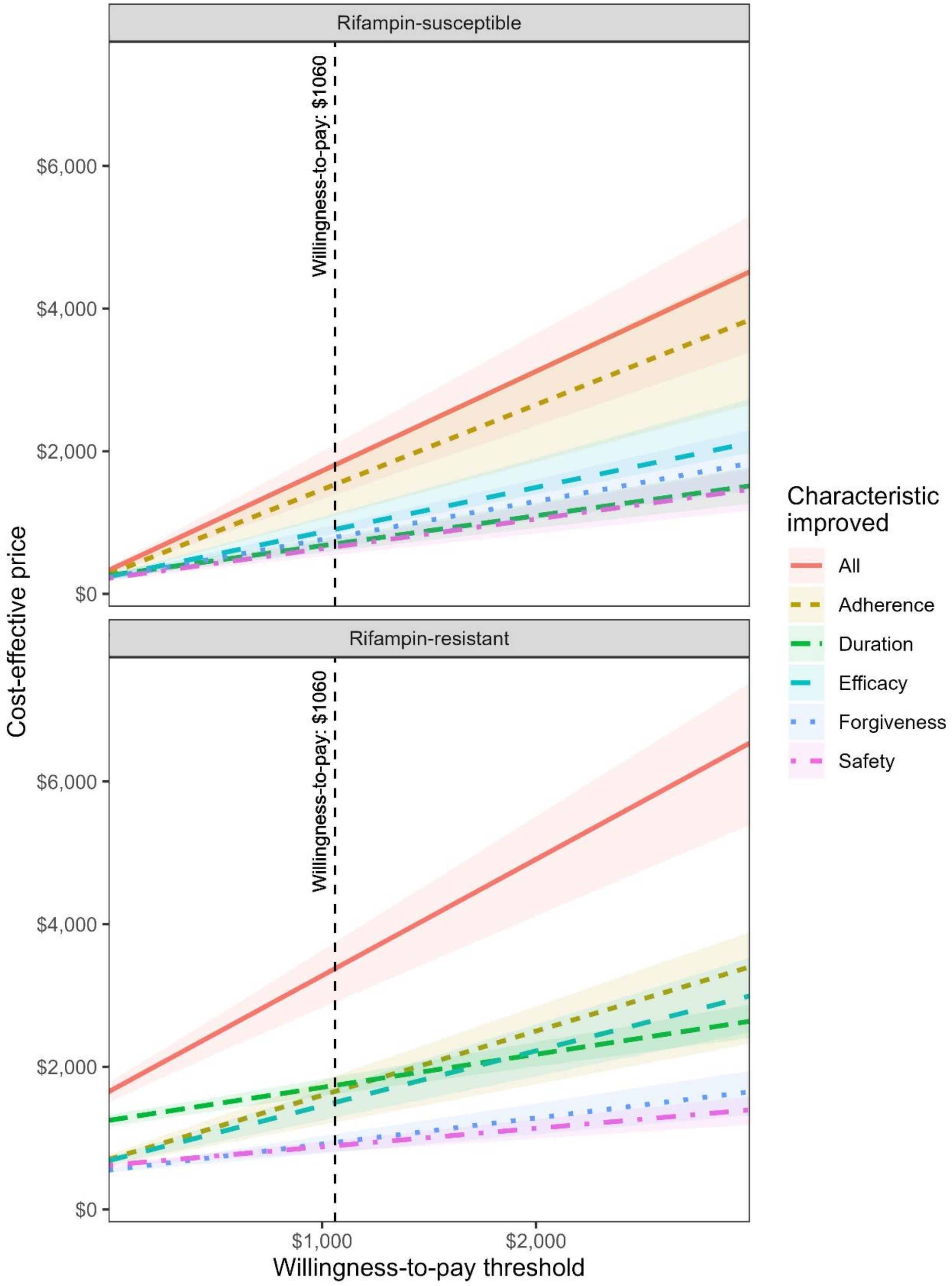

Figure shows how the cost-effective price thresholds (y-axis) for TRP-minimal rifampin-susceptible (RS) and rifampin-resistant (RR) regimens in the Philippines vary with the willingness-to-pay threshold (x-axis) when 1 characteristic at a time is set to its "optimal" value in the TRPs (yellow through purple), and when all characteristics are set to their "optimal" values (red="TRP-Optimal" regimen). The willingness-to-pay threshold used in the main analysis is shown with a dashed vertical line. Shaded areas indicate 95% uncertainty intervals around the means.

Figure S9: Cost-effective prices with variation in willingness-to-pay: Philippines, health systems perspective

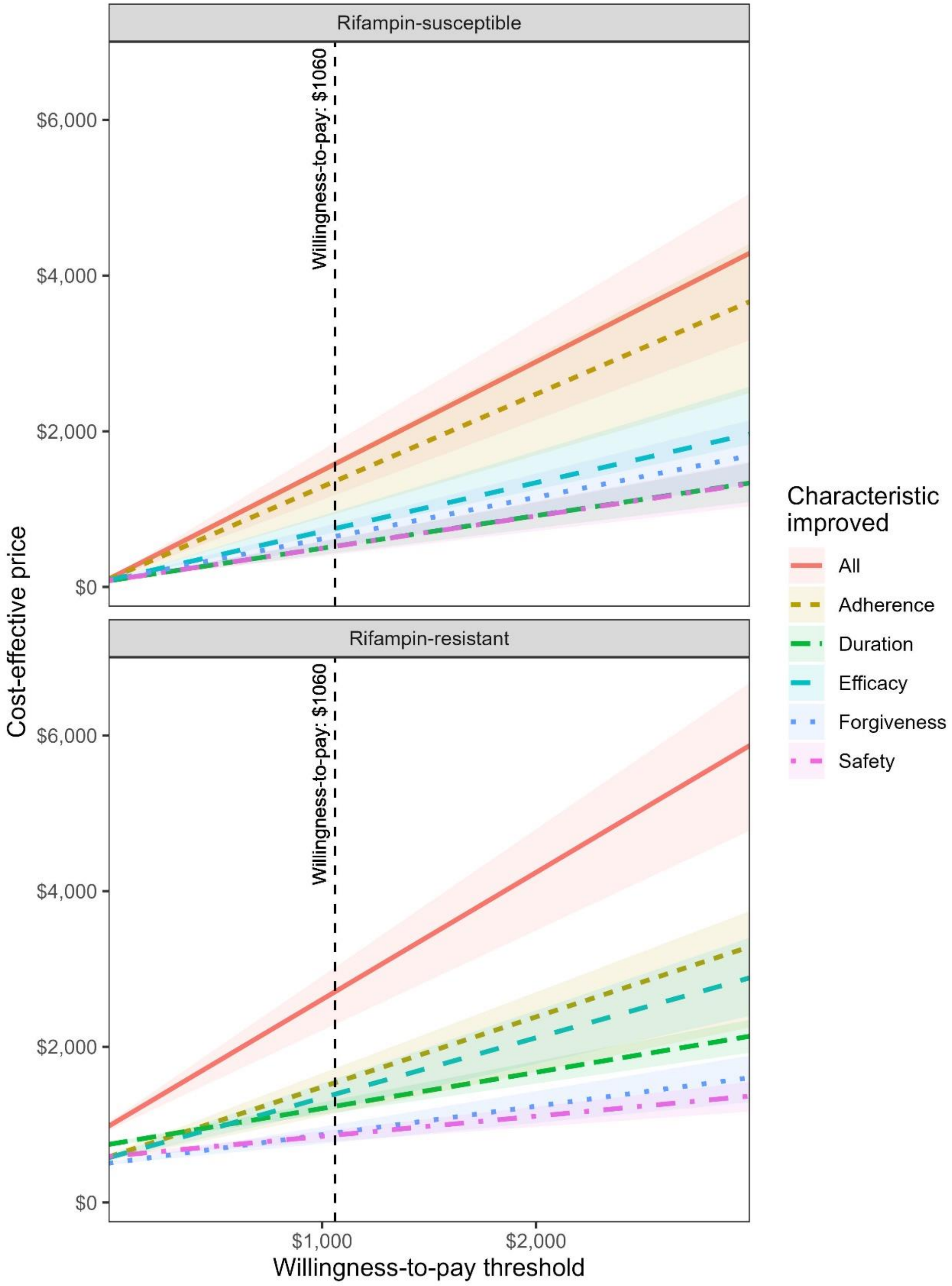

Figure shows how the cost-effective price thresholds (y-axis) for TRP-minimal rifampin-susceptible (RS) and rifampin-resistant (RR) regimens in the Philippines vary with the willingness-to-pay threshold (x-axis) when 1 characteristic at a time is set to its “optimal” value in the TRPs (yellow through purple), and when all characteristics are set to their “optimal” values (red=”TRP-Optimal” regimen). The willingness-to-pay threshold used in the main analysis is shown with a dashed vertical line. Shaded areas indicate 95% uncertainty intervals around the means.
